# Establishment of the TBX-code reveals aberrantly activated T-box gene TBX3 in Hodgkin lymphoma

**DOI:** 10.1371/journal.pone.0259674

**Published:** 2021-11-22

**Authors:** Stefan Nagel, Corinna Meyer

**Affiliations:** Department of Human and Animal Cell Lines, Leibniz-Institute DSMZ–German Collection of Microorganisms and Cell Cultures, Braunschweig, Germany; Albany Medical College, UNITED STATES

## Abstract

T-box genes encode transcription factors which control basic processes in development of several tissues including cell differentiation in the hematopoietic system. Here, we analyzed the physiological activities of all 17 human T-box genes in early hematopoiesis and in lymphopoiesis including developing and mature B-cells, T-cells, natural killer (NK)-cells and innate lymphoid cells. The resultant expression pattern comprised six genes, namely EOMES, MGA, TBX1, TBX10, TBX19 and TBX21. We termed this gene signature TBX-code which enables discrimination of normal and aberrant activities of T-box genes in lymphoid malignancies. Accordingly, expression analysis of T-box genes in Hodgkin lymphoma (HL) patients using a public profiling dataset revealed overexpression of EOMES, TBX1, TBX2, TBX3, TBX10, TBX19, TBX21 and TBXT while MGA showed aberrant downregulation. Analysis of T-cell acute lymphoid leukemia patients indicated aberrant overexpression of six T-box genes while no deregulated T-box genes were detected in anaplastic large cell lymphoma patients. As a paradigm we focused on TBX3 which was ectopically activated in about 6% of HL patients analyzed. Normally, TBX3 is expressed in tissues like lung, adrenal gland and retina but not in hematopoiesis. HL cell line KM-H2 expressed enhanced TBX3 levels and was used as an *in vitro* model to identify upstream regulators and downstream targets in this malignancy. Genomic studies of this cell line showed focal amplification of the TBX3 locus at 12q24 which may underlie its aberrant expression. In addition, promoter analysis and comparative expression profiling of HL cell lines followed by knockdown experiments revealed overexpressed transcription factors E2F4 and FOXC1 and chromatin modulator KDM2B as functional activators. Furthermore, we identified repressed target genes of TBX3 in HL including CDKN2A, NFKBIB and CD19, indicating its respective oncogenic function in proliferation, NFkB-signaling and B-cell differentiation. Taken together, we have revealed a lymphoid TBX-code and used it to identify an aberrant network around deregulated T-box gene TBX3 in HL which promotes hallmark aberrations of this disease. These findings provide a framework for future studies to evaluate deregulated T-box genes in lymphoid malignancies.

## Introduction

Lymphopoiesis encompasses the generation of all types of lymphocytes and starts with hematopoietic stem cell (HSC)-derived common lymphoid progenitors (CLP) in the bone marrow. According developmental processes are regulated mainly at the transcriptional level and specific transcription factors (TFs) control the development of lymphoid lineages [1–4]: for example, PAX5 and TCF3 in B-cells, BCL11B and GATA3 in T-cells, ID2 and NFIL3 in NK-cells, and GATA3, TBX21 and TCF7 in innate lymphoid cells (ILCs). In B-cell development, several TFs, like BCL6, EBF1, NKX6-3 and PAX5 form a regulatory network to control basic differentiation operations [5–7]. Deregulation of these TFs contributes to the generation of B-cell malignancies, highlighting their pathogenic potential. Chromosomal rearrangements, gene mutations or virus infection have been reported to underlie their aberrant expression [8–10]. Therefore, the study of developmental TFs promotes our understanding of both normal lymphopoiesis and lymphoid tumorigenesis.

TFs are classified systematically according to similarities in sequence and structure [11]. Homeobox and T-box genes encode two main groups of TFs operating in basic developmental processes whose mutation or deregulation causes developmental diseases or cancer [12–14]. Each group shares a particular conserved domain which performs sequence-specific DNA-binding and protein interactions with cofactors and chromatin. The homeodomain consists of 60 amino acid residues creating three alpha-helices separated by two loops. The third helix interacts with the major groove of the DNA molecule to confer contact specificity [15]. Homeobox genes fall into eleven classes and several subclasses according to sequence similarities in their conserved homeobox [16]. In contrast, the T-box domain consists of about 180 amino acid residues creating a seven-stranded beta-barrel structure belonging to the s-type immunoglobulin domain class [17]. T-box proteins interact with the major and minor grooves of DNA as monomer or dimer [17]. A consensus DNA binding site for all T-box proteins has been identified and called T-element [18]. Thus, homeodomain and T-box proteins differ in sequence and structure of their DNA-binding domains but share potency in the control of cell differentiation and tissue development.

The clustered homeobox genes belong to the HOXL subclass and are expressed within the head and branchial region of the embryo in a particular pattern termed HOX-code [19]. Throughout the recent years, we outlined the NKL-code which details the normal expression patterns of NKL subclass homeobox genes in the course of blood cell development covering early hematopoiesis, myelopoiesis and lymphopoiesis [7,20–23]. Furthermore, we have also described the TALE-code showing the physiological signature of the TALE class homeobox genes in lymphopoiesis [24]. Gene signatures like the HOX-, NKL- or TALE-code are generated by closely related homeobox genes performing similar functions and operating in particular tissue compartments. They allow the detection and evaluation of deregulated genes in cancer patients which might be useful for diagnostics and even eventually therapeutics [13].

Here, we followed the same approach of expression analysis used for the generation of the lymphoid NKL- and TALE-codes for the study of T-box genes in early hematopoiesis and in lymphopoiesis. The human genome contains 17 T-box genes which are arranged in five subfamilies: namely T (containing TBXT and TBX19), Tbx1 (TBX1, TBX10, TBX15, TBX18, TBX20, TBX22), Tbx2 (TBX2, TBX3, TBX4, TBX5), Tbx6 (TBX6, MGA) and Tbr1 (EOMES, TBX21, TBR1). This classification reflects the evolutionary history of these genes in metazoa [25]. Some members have been intensively investigated and reported to regulate differentiation and function of blood cells including TBX21 (also known as Tbet) in B-cells, NK-cells, T-cells and ILCs [26–29] and EOMES in T-cells, NK-cells and ILC1 [29–34]. Interestingly, TBX21 and EOMES are members of the same subfamily, Tbr1. However, the role of the remaining T-box genes in hematopoiesis is largely unknown. Our approach revealed six T-box genes specifically expressed in early hematopoiesis and lymphopoiesis. We termed the resultant physiological expression pattern “TBX-code”, which assisted identification of deregulated T-box genes in lymphoid malignancies including T-cell acute lymphoid leukemia (T-ALL), anaplastic large cell lymphoma (ALCL), and Hodgkin lymphoma (HL).

T-ALL cells are derived from developing thymic T-cells while the origin of the malignant ALCL cells is less clear but they may derive from innate lymphoid cell type 3 (ILC3) [35,36]. Hodgkin and Reed-Sternberg (HRS) cells, the presumed malignant cells in HL, derive from developing germinal centre B-cells [37]. Several TFs involved in B-cell development are downregulated in HRS cells resulting in incomplete cell differentiation [38–40]. Aberrantly activated NFkB-signalling and subsequent inhibition of apoptosis represent additional hallmarks of this malignancy [41]. Here, we exploited the TBX-code established in this study and revealed an aberrant gene regulatory network centered around ectopically expressed TBX3 probably implicated in the pathogenesis of HL.

## Materials and methods

### Expression profiling and RNA-seq data analysis

For our screening approach of normal cell types we exploited both expression profiling and RNA-sequencing data which were obtained from Gene Expression Omnibus (GEO, www.ncbi.nlm.nih.gov) as described previously [7,20–23]. To analyze patient samples from HL, ALCL and T-ALL we used the public datasets GSE12453, GSE14879 and GSE26713, respectively [42–44]. Gene expression profiling data for HL cell lines were generated and compared as reported previously (GSE115191) [45]. RNA-seq data from 100 leukemia/lymphoma cell lines (termed LL-100) including HL are available at ArrayExpress (www.ebi.ac.uk/arrayexpress) via E-MTAB-7721 [46]. Corresponding gene expression values were visualized using shinyNGS (https://github.com/pinin4fjords/shinyngs).

### Cell lines and treatments

Cell lines were held by the DSMZ (Braunschweig, Germany) and cultivated as described previously [47]. All cell lines had been authenticated and tested negative for mycoplasma infection. Modification of gene expression levels was performed using gene specific siRNA oligonucleotides with reference to AllStars negative Control siRNA (siCTR) obtained from Qiagen (Hilden, Germany). The gene expression constructs for TBX3, E2F4 and an empty control-vector were obtained from Origene (Wiesbaden, Germany). SiRNAs (80 pmol) and vector DNA (2 μg) were transfected into 1x10^6^ cells by electroporation using the EPI-2500 impulse generator (Fischer, Heidelberg, Germany) at 350 V for 10 ms. Electroporated cells were harvested after 20 h cultivation. Trichostatin A (TSA) was obtained from Sigma (Taufkirchen, Germany) and added for 20 h at a final concentration of 10 μg/ml.

Proliferation and apoptosis were analyzed using the IncuCyte S3 Live-Cell Analysis System (Essen Bioscience, Hertfordshire, UK). For detection of apoptotic cells we used the IncuCyte Caspase-3/7 Green Apoptosis Assay diluted at 1:2000 (Essen Bioscience). Live-cell imaging experiments were performed twice with fourfold parallel tests. Statistical significance was calculated for the last timepoint by T-Test.

### Polymerase chain-reaction (PCR) analyses

Total RNA was extracted from cultivated cell lines using TRIzol reagent (Invitrogen, Darmstadt, Germany). Primary human total RNA derived from spleen, lung and retina was purchased from Biochain/BioCat (Heidelberg, Germany), and RNA from peripheral CD19-positive B-cells and CD3-positive T-cells from Miltenyi Biotec (Bergisch Gladbach, Germany). cDNA was synthesized using 5 μg RNA, random priming and Superscript II (Invitrogen). Real time quantitative (RQ)-PCR analysis was performed using the 7500 Real-time System and commercial buffer and primer sets (Applied Biosystems/Life Technologies, Darmstadt, Germany). For normalization of expression levels we quantified the transcripts of TATA box binding protein (TBP).

For copy number quantification we extracted genomic DNA using the High Pure PCR Template Preparation Kit (Roche Diagnostics, Mannheim, Germany). Analyses of TBX3 and TBX5 gene copy numbers were performed with reference to the MEF2C control. The following oligonucleotides (obtained from Eurofins MWG, Ebersberg, Germany) were used: TBX3-for 5`-GAGCCTCTCCATGAGAGATCCG-3´, TBX3-rev 5´-TGATCCATGATCGGCTTGGCCAGG-3´, TBX5-for 5`-AGCGCACAGCAGAGGAGGTGTG-3´, TBX5-rev 5´-GCTGCTCCTAGCAGGGAAGCCG-3´, MEF2C-for 5´-GCAGGAATTTGGGAACTGAG-3´, MEF2C-rev 5´-CCCATAGTCCCCGTTTTTCT-3´.

Quantitative analyses were performed as biological replicates and measured in triplicate. Standard deviations are presented in the figures as error bars. Statistical significance was assessed by Student´s T-Test (two-tailed) and the calculated p-values indicated by asterisks (* p<0.05, ** p<0.01, *** p<0.001, n.s. not significant).

### Protein analysis

Western blots were generated by the semi-dry method. Protein lysates from cell lines were prepared using SIGMAFast protease inhibitor cocktail (Sigma). Proteins were transferred onto nitrocellulose membranes (Bio-Rad, München, Germany) and blocked with 5% dry milk powder dissolved in phosphate-buffered-saline buffer (PBS). The following antibodies were used: alpha-Tubulin (Sigma, #T6199), TBX3 (Thermo Fisher, #711087), E2F4 (Cell Signaling Technology, #40291), BCL2L1 (Cell Signaling, #2764), and CDKN2A (Abcam, #ab108349). For loading control blots were reversibly stained with Poinceau (Sigma) and detection of alpha-Tubulin (TUBA) performed thereafter. Secondary antibodies were linked to peroxidase for detection by Western-Lightning-ECL (Perkin Elmer, Waltham, MA, USA). Documentation was performed using the digital system ChemoStar Imager (INTAS, Göttingen, Germany).

Immuno-cytology was performed as follows: cells were spun onto slides and subsequently air-dried and fixed with methanol/acetic acid for 90 s. Antibodies were diluted 1:20 in PBS containing 5% BSA and incubated for 30 min. Washing was performed 3 times with PBS. Preparations were incubated with secondary antibody (diluted 1:100) for 20 min. After final washing cells were mounted for nuclear in Vectashield (Vector Laboratories, Burlingame, CA), containing DAPI. Documentation of subcellular protein localization was performed using an Axio-Imager microscope (Zeiss, Göttingen, Germany) configured to a dual Spectral Imaging system (Applied Spectral Imaging, Neckarhausen, Germany).

Flow cytometry was performed for quantification of CD19 protein on the cell surface. Cells were washed and incubated with mouse anti-human CD19 mAB (BD Biosciences, Heidelberg, Germany) or with the isotope-matched control mouse immunoglobulin (BD Biosciences) for 30 min at 4°C. Subsequently, cells were treated with FITC conjugated anti-mouse secondary antibody (Biozol, Eching, Germany) and Propidium Iodide (Sigma). Labeled cells were analyzed on a FACS Calibur (BD Biosciences) using CellQuest Pro software.

### Genomic analyses

For genomic profiling genomic DNA of HL cell lines was prepared by the Qiagen Gentra Puregene Kit (Qiagen). Labelling, hybridization and scanning of Cytoscan HD arrays was performed by the Genome Analytics Facility located at the Helmholtz Centre for Infection Research (Braunschweig, Germany), according to the manufacturer´s protocols (Affymetrix, High Wycombe, UK). Data were interpreted using the Chromosome Analysis Suite software version 3.1.0.15 (Affymetrix) and copy number alterations determined accordingly.

### Reporter-gene assay

For creation of reporter gene constructs we combined a reporter with a regulatory genomic fragment derived from the upstream region of CDKN2A. PCR products of the corresponding genomic region (regulator) and of the HOXA9 gene, comprising exon1-intron1-exon2 (reporter), were cloned into respective *Hind*III/*Bam*HI and *Eco*RI sites of expression vector pcDNA3 downstream of the CMV enhancer. Oligonucleotides used for the amplification of the regulator were obtained from Eurofins MWG. Their sequences were as follows: CDKN2A-for 5´-CTAAGCTTCCTGAGTAGCTGGAATTACACACG-3´, CDKN2A-rev 5´-TAGGATCCGTGCCACACATCCTAAGCTAATAC-3´. Introduced restriction sites used for cloning are underlined. Constructs were validated by sequence analysis (Eurofins MWG). Transfections of plasmid-DNA into NIH-3T3 cells (DSMZ) were performed using SuperFect Transfection Reagent (Qiagen). Commercial HOXA9 and TBP assays were used for RQ-PCR to quantify the spliced reporter-transcript, corresponding to the regulator activity (Thermo Fisher Scientific).

## Results

### Normal T-box gene activities in lymphopoiesis are called TBX-code

**Fig 1:**
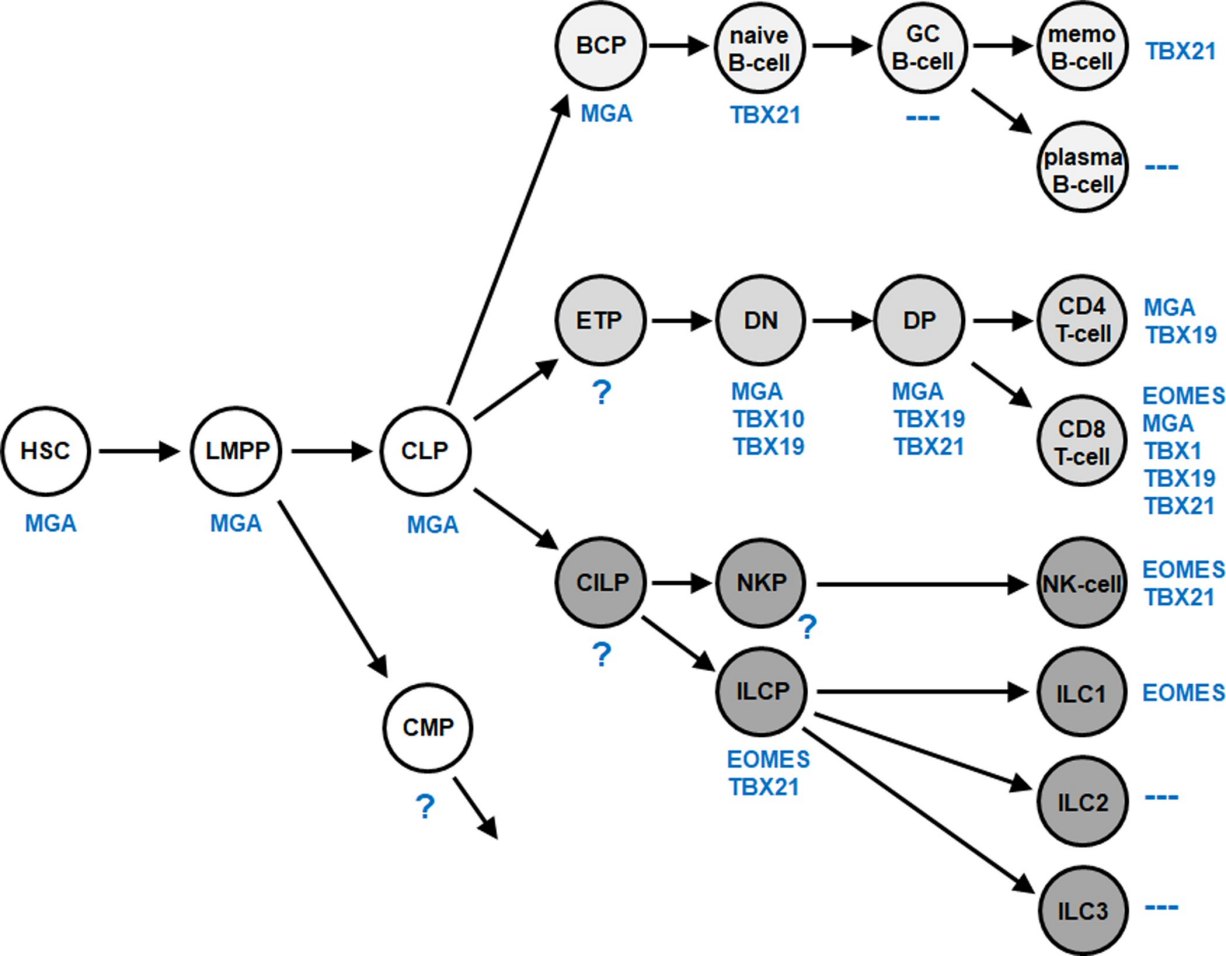
Lymphoid TBX-code. This diagram summarizes screening results for T-box gene expression (blue) in early hematopoiesis and lymphopoiesis. We termed this expression pattern TBX-code. Of note, TBX3 is not expressed in these hematopoietic entities. BCP: B-cell progenitor, CILP: Common innate lymphoid progenitor, CLP: Common lymphoid progenitor, CMP: Common myeloid progenitor, DN: Double negative T-cell, DP: Double positive T-cell, ETP: Early T-cell progenitor, GC: Germinal center, HSC: Hematopoietic stem cell, ILC: Innate lymphoid cell, ILCP: Innate lymphoid cell progenitor, LMPP: Lymphoid and myeloid primed progenitor, memo: Memory, NKP: NK-cell progenitor.

To identify normal expression patterns for all 17 human T-box genes in early hematopoiesis and lymphopoiesis several public datasets were analyzed. Dataset GSE69239 contains RNA-seq data for HSCs, lymphoid and myeloid primed progenitors (LMPP), CLP, B-cell progenitors (BCP) and T-cell progenitors of the double negative (DN) and double positive (DP) stages in addition to mature single positive CD4+ and CD8+ T-cells [48]. Stages of B-cell development were analyzed using gene expression profiling dataset GSE56315, and mature lymphocytes isolated from peripheral blood via dataset GSE72642 [49,50]. ILC progenitors (ILCP) in addition to three types of differentiated ILCs, namely ILC1, ILC2 and ILC3, were examined using RNA-seq datasets GSE124474 and E-MTAB-8494 [51,52]. To discriminate positive and negative expression levels, cutoffs were adopted from our previous studies [7,20–23]. The screening results are shown in **S1 Fig** and summarized in **Fig 1**. We detected expression of six T-box genes in the hematopoietic entities analyzed, comprising EOMES, MGA, TBX1, TBX10, TBX19 and TBX21, and termed the combined T-box gene signature TBX-code.

We found T-box gene activities in all lymphoid lineages. The TBX-code contains genes from all T-box subfamilies except Tbx2. The numbers of expressed genes in single entities ranged from zero to five. Stem and early progentor cells expressed MGA only. The B-cell lineage showed MGA and TBX21 activities. The greatest number of expressed T-box genes was detected in the T-cell lineage and included all six members of the TBX-code. Mature NK-cells and ILCPs expressed TBX21 and EOMES, ILC1 only EOMES while no T-box gene activities were detected in ILC2 and ILC3. Thus, T-box genes show a specific expression pattern which may underlie regulation of normal cell differentiation in lymphopoiesis. By the same token, aberrations of this TBX-code may promote generation of leukemia and lymphoma.

### Aberrant T-box gene activities in lymphoid malignancies

Next, we proceeded to analyze expression of all T-box genes in patients with diverse lymphoid malignancies for comparison with the newly established TBX-code. Public dataset GSE12453 contains gene expression profiling data of 12 classical and 5 nodular lymphocyte predominant HL patients and of normal developing B-cells used as controls [42]. This comparison revealed nine deregulated T-box genes in HL patients, comprising overexpressed EOMES, TBX1, TBX2, TBX3, TBX10, TBX19, TBX21 and TBXT and downregulated MGA (**S2A Fig and Table 1**). Furthermore, we analyzed T-box gene deregulation in 117 T-ALL and 16 ALCL patients using public datasets GSE26713 and GSE14879, respectively [43,44]. In T-ALL patients we found six aberrantly overexpressed genes, namely MGA, TBX1, TBX3, TBX6, TBX19 and TBX21 while no deregulated genes were detected in ALCL patients (**S2B and S2C Fig and Table 1**). Thus, our T-box gene expression results show significant differences between lymphoid malignancies, indicating that deregulated T-box genes may play a role in the pathogenesis of HL and T-ALL but not of ALCL.

**Fig 2:**
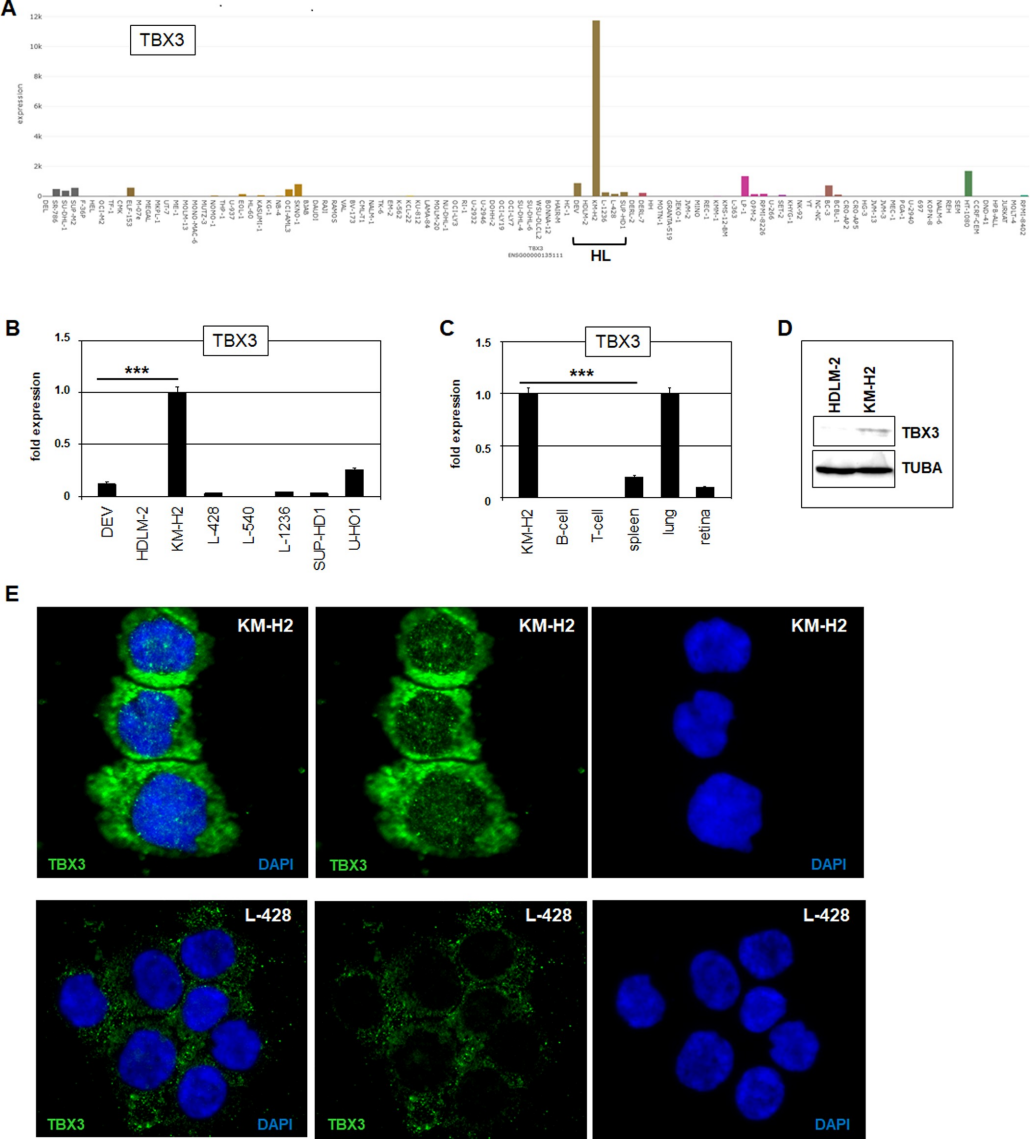
TBX3 and TBX5 expression in HL cell lines. (A) RNA-seq data show expression levels of TBX3 in 100 leukemia/lymphoma cell lines using dataset E-MTAB-7721. Note the singularly high transcript levels in HL cell line KM-H2. HL cell lines are indicated. (B) RQ-PCR analysis of TBX3 in HL cell lines, confirming elevated expression in KM-H2. (C) RQ-PCR analysis of TBX3 in KM-H2 in comparison to primary samples derived from B-cells, T-cells, spleen, lung and retina. (D) Western blot analysis of TBX3 in HL cell lines shows TBX3 expression at the protein level in KM-H2. (E) Immuno-fluorescence microscopy of KM-H2 (above) and L-428 cells (below) using TBX3 antibody (green) and nuclear counterstain DAPI (blue).

**Table 1:** T-box gene activity in HL, T-ALL and ALCL.

T-box gene	T-box gene	TBX-code	HL patients		HL cell lines	T-ALL patients	ALCL patients
subfamily	name	members	GSE12453 (N = 17)		E-MTAB-7721 (N = 6)	GSE26713 (N = 117)	GSE14879 (N = 16)
Tbx1	TBX1	TBX1	TBX1	up	-	TBX1 up	-
	TBX10	TBX10	TBX10	up	-	-	-
	TBX15	-	-	-	-	-	-
	TBX18	-	-	-	SUP-HD1	-	-
	TBX20	-	-	-	-	-	-
	TBX22	-	-	-	-	-	-
Tbx2	TBX2	-	TBX2	up	-	-	-
	TBX3	-	TBX3	up	KM-H2	TBX3 up	-
	TBX4	-	-	-	-	-	-
	TBX5	-	-	up	KM-H2	-	-
Tbx6	TBX6	-	-	-	-	TBX6 up	
	MGA	MGA	MGA	down	-	MGA up	-
Tbr1	EOMES	EOMES	EOMES	up	L-428, L-1236	-	-
	TBX21	TBX21	TBX21	up	-	TBX21 up	-
	TBR1	-	-	-	-	-	-
T	TBXT	-	TBXT	up	L-1236	-	-
	TBX19	TBX19	TBX19	up	HDLM-2, L-1236	TBX19 up	-
5	17	6	9			6	0

All 17 human T-box genes are ordered according to their classification in five subfamilies. TBX-code members are indicated, and T-box gene activities are given for HL patients and HL cell lines, T-ALL patients, and ALCL patients.

### TBX3 is overexpressed in HL cell line KM-H2

Accordingly, we then focused on the role of deregulated T-box genes in HL. Expression analysis of six HL cell lines employing LL-100 RNA-seq data (E-MTAB-7721) demonstrated overexpression of EOMES in L-428 and L-1236, TBX3 and TBX5 in KM-H2, TBX18 in SUP-HD1, TBX19 in HDLM-2 and L-1236, and TBXT in L-1236 (**S3 Fig and Table 1**). Thus, the most aberrantly expressed T-box genes detected in HL patients were also conspicuously overexpressed in HL cell lines. Of these, TBX3 belongs to the subfamily Tbx2, whose members are hitherto unrepresented in the TBX-code. TBX3 is an oncogene reported in solid tumors, including breast cancer, fibrosarcoma, melanoma and lung cancer but not as yet in hematopoietic neoplasms [53,54]. To substantiate its possible oncogenic role thus implied in hemic neoplasia, we investigated mechanisms underlying its pathogenic deregulation in HL using cell line KM-H2 as model.

KM-H2 expressed elevated levels of TBX3 as indicated by RNA-seq data and confirmed by RQ-PCR (**Fig 2A and 2B**). Comparison of TBX3 transcript levels from KM-H2 with representative primary samples demonstrated a similar gene activity in lung and lower activities in spleen and retina. As shown by our screening data, no TBX3 expression was detected by RQ-PCR in primary peripheral B- and T-cells from healthy donors (**Fig 2C**). Western blot and immuno-fluorescence-analysis confirmed TBX3 expression in KM-H2 at the protein level (**Fig 2D and 2E**). At the subcellular level TBX3 was detected in nuclear speckles and uniform in the cytoplasma, suggesting that transcriptional activity of TBX3 is restricted to specific genes and may be controlled by nuclear import.

### TBX3 is activated by genomic amplification and particular oncogenes

**Fig 3:**
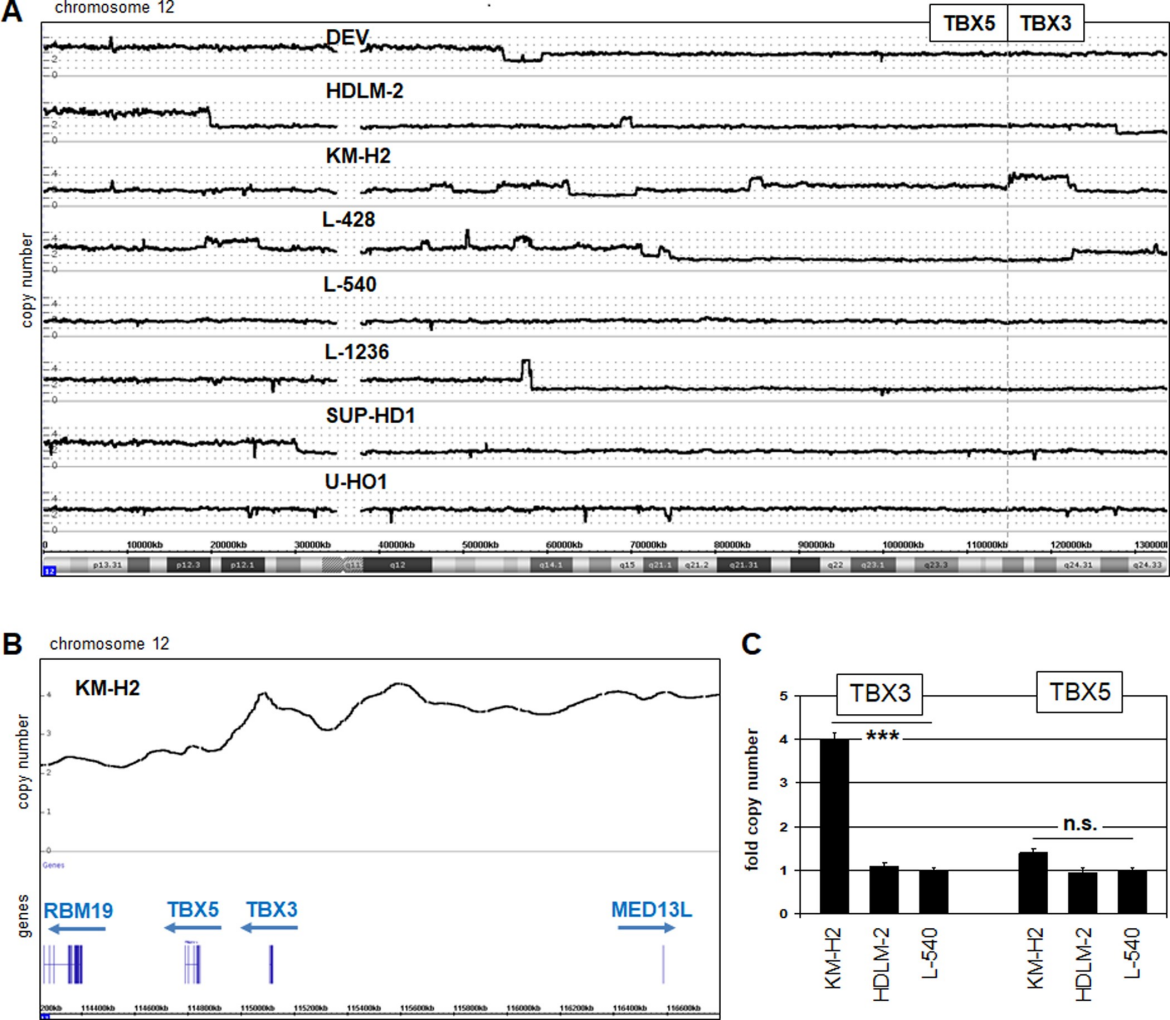
Genomic analyses of the TBX3 and TBX5 locus. (A) Genomic profiling data for chromosome 12 of eight HL cell lines. The neighboring gene loci of TBX3 and TBX5 are indicated, revealing amplification in KM-H2. (B) Enlarged picture of genomic profiling data for KM-H2, showing amplification of TBX3 but not of TBX5. Genomic Q-PCR analysis of HL cell line KM-H2 in comparison to HDLM-2 and L-540 confirmed amplification of the TBX3 gene in KM-H2.

KM-H2 cells overexpressed both TBX3 and TBX5, indicating the possible mechanistic connection of their activation although the expression level of TBX3 strongly exceeded TBX5 (**Fig 2A**). TBX3 is located together with its gene neighbor TBX5 at chromosomal position 12q24 in a tandem arrangement, reflecting their evolutionary history [25]. Chromosomal and genomic aberrations widely contribute to gene deregulation in cancer including HL. Accordingly, the reported karyotype of KM-H2 which is highly rearranged, was updated from that reported previously [24]: human hypotriploid, 59(56–60)<3n>der(X)der(Y),–X, -1, -1,add(1)(q32), -2, -2, -3, add(3)(q26), add(3)(q26), -4, del(4)(q26q31), i(4)(q10), -5, -5, add(5)(p11), -6, add(6)(q14), add(7)(p14), add(7)(q34), del(7)(q31), -8, add(8)(q24), del(8)(p12), -10, -10, -10, -11, del(11)(q23), -13, i(13)(q10), der(13)t(11;13)(p13;q13), -14, add(14)(p12)x2, -15, -15, -16, -16, del(17)(p12), -18, i(18)(q10), -19, -20, +25mar, including several with HSR regions associated with gene amplification. The absence of overt rearrangements at 12q24 prompted closer examination of this locus by genomic profiling to quantify copy numbers. Accordingly, genomic array data for chromosome 12 from KM-H2 were analyzed in comparison to HL control cell lines DEV, HDLM-2, L-428, L-540, L-1236, SUP-HD1, and U-HO1. Only in KM-H2 was amplification of TBX3 and TBX5 detected (**Fig 3A and 3B**). Genomic Q-PCR analysis confirmed this gain which was significantly higher for TBX3 than TBX5 (**Fig 3C**). Thus, the aberrant expression levels observed for TBX3 and TBX5 reflected their copy number gains which, thus, represent a major oncogenic mechanism of activation in KM-H2.

**Fig 4:**
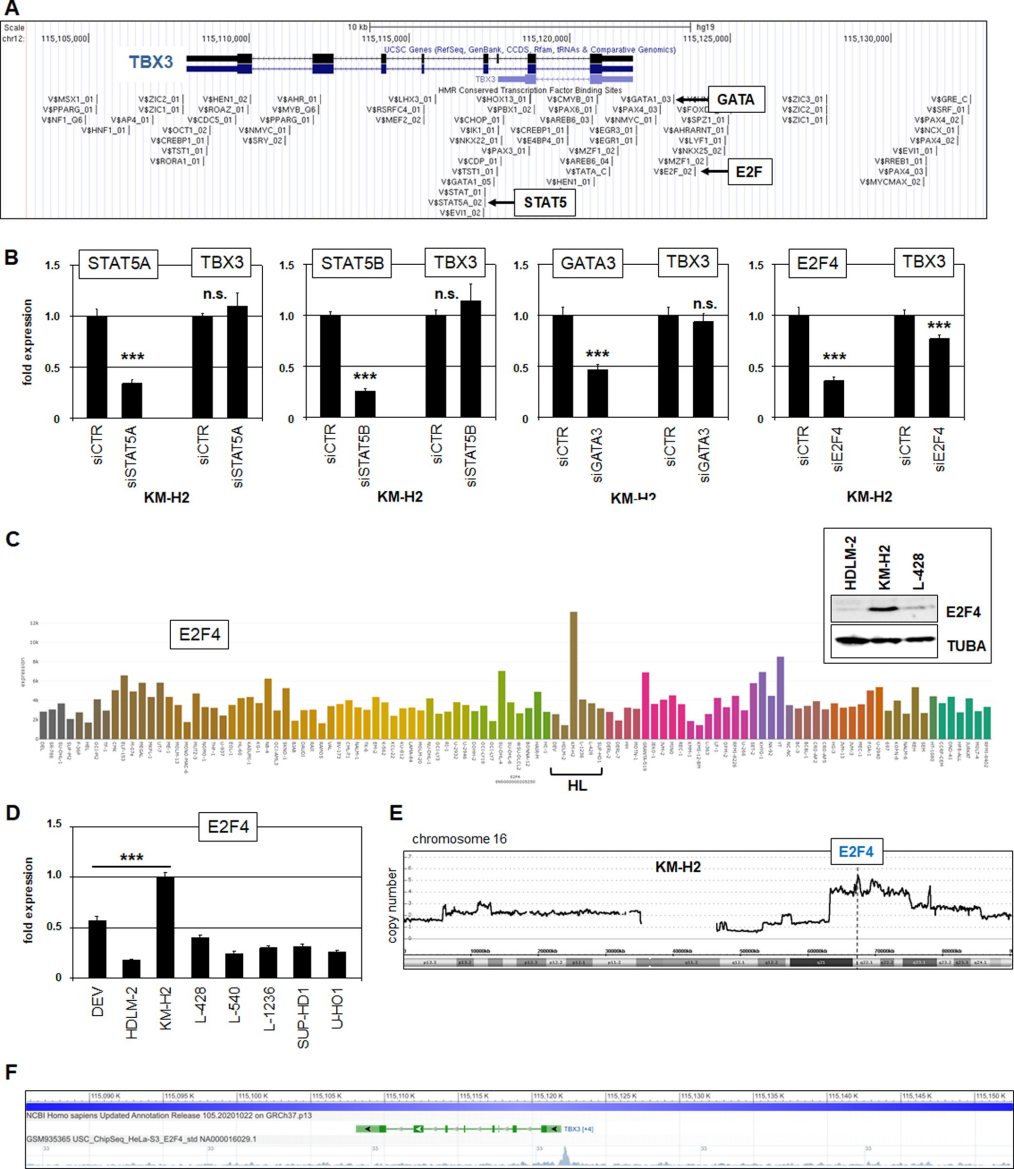
TBX3 promoter analysis. (A) Potential TF binding sites at the TBX3 locus obtained from the UCSC genome browser (hg19). Binding sites for the TFs STAT5, GATA and E2F are highlighted. (B) RQ-PCR analysis of TBX3 after siRNA-mediated knockdown of STAT5A, STAT5B, GATA3, and E2F4. The data indicated that E2F4 activated TBX3 while STAT5A, STAT5B and GATA3 showed no transcriptional impact. (C) RNA-seq data show expression levels of E2F4 in 100 leukemia/lymphoma cell lines using dataset E-MTAB-7721. Western blot analysis of E2F4 protein in HL cell lines HDLM-2, KM-H2 and L-428, demonstrating high levels in KM-H2. TUBA served as loading control (insert). (D) RQ-PCR analysis of E2F4 expression in HL cell lines, confirming elevated transcript levels in KM-H2. (E) Genomic profiling data for chromosome 16 of HL cell line KM-H2, demonstrating amplification of E2F4 at 16q22. (F) ChIP data from ENCODE for E2F4 in HELA-S3 cells demonstrating binding at the TBX3 promoter.

To identify TFs involved in TBX3 activation we screened its promoter region for potential binding sites using the UCSC genome browser (**Fig 4A**). The TBX3 gene in addition to its 10 kb upstream region contain several TF binding sites including those for STAT5, GATA3 and E2F. These TFs have been shown to play an oncogenic role in HL and were, therefore, selected for more detailed analysis [55–57]. SiRNA-mediated knockdowns were performed in KM-H2 cells and subsequently tested by RQ-PCR analysis. These analyses discounted TBX3 regulation by STAT5A, STAT5B and GATA3 while E2F4 operated as activator (**Fig 4B**). E2F4 showed elevated expression levels in KM-H2 as demonstrated by RNA-seq data and confirmed by RQ-PCR and Western blot analysis (**Fig 4C and 4D**). Moreover, although the locus of E2F4 at 16q22 escaped chromosomal rearrangement (see above) it was also amplified in KM-H2 (**Fig 4E**), indicating its likely activation by genomic aberration. Finally, public chromatin immuno-precipitation (ChIP) data from the ENCODE project show binding of E2F4 at TBX3 (**Fig 4F**), supporting its direct impact in transcriptional regulation.

**Fig 5:**
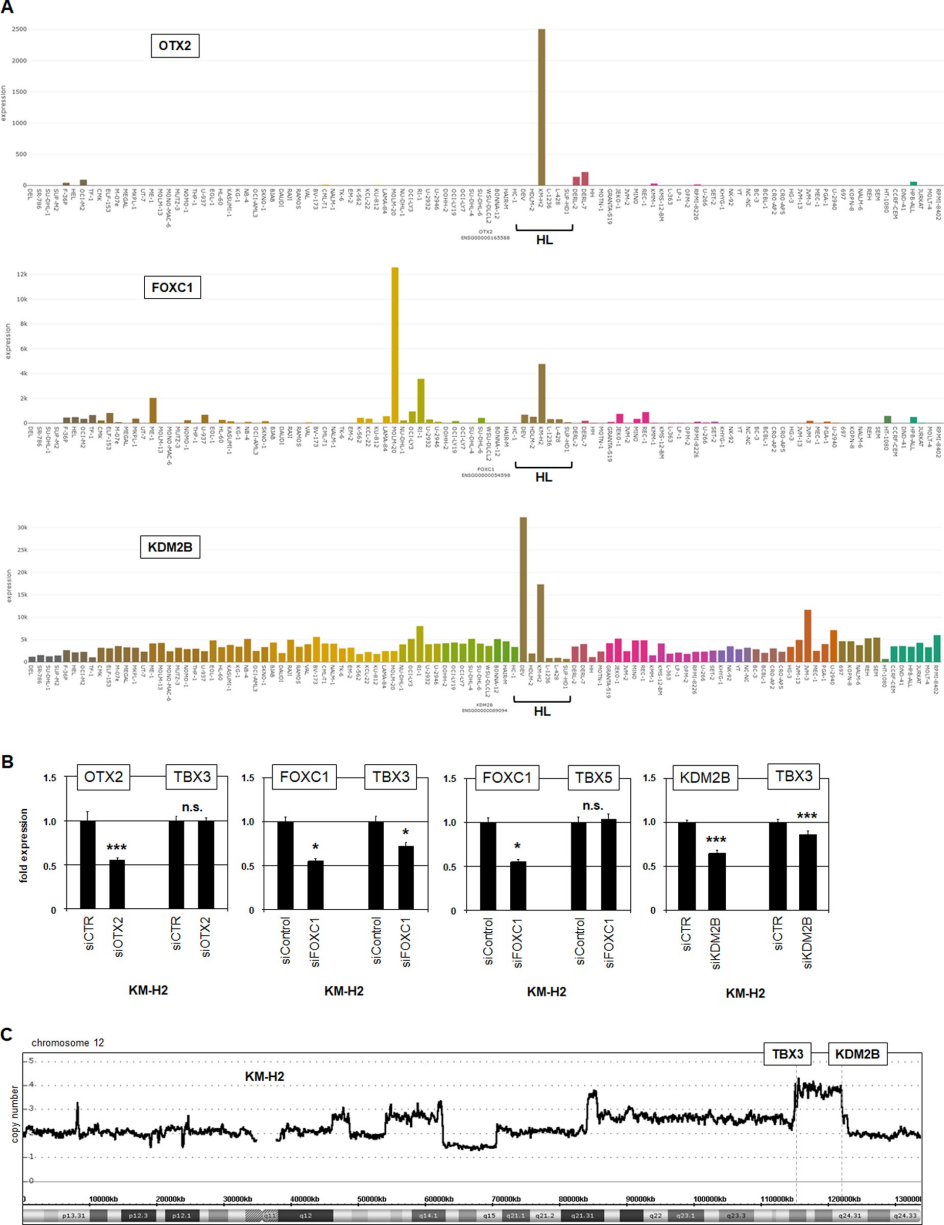
FOXC1 and KDM2B activate TBX3 expression. (A) RNA-seq data from 100 leukemia/lymphoma cell lines (dataset E-MTAB-7721) showing expression of OTX2 (above), FOXC1 (middle), and KDM2B (below). HL cell lines are indicated. Note high transcript levels in HL cell line KM-H2. (B) RQ-PCR analysis of TBX3 and TBX5 after siRNA-mediated knockdown of OTX2, FOXC1, and KDM2B. Data indicated that FOXC1 and KDM2B activated TBX3 while discounting transcriptional impact of OTX2. (C) Genomic profiling data for chromosome 12 of HL cell line KM-H2, demonstrating amplification of TBX3 and KDM2B at 12q24.

To identify additional activators of TBX3 we performed comparative expression profiling analysis of HL cell lines. We compared KM-H2 versus DEV, HDLM-2, L-428, L-540, L-1236, SUP-HD1 and U-HO1, to reveal differentially expressed genes (**S1 Table**). Among the top-1000 overexpressed genes in KM-H2 we detected in addition to TBX3 and TBX5 the genes OTX2, FOXC1 and KDM2B respectively encoding two oncogenic TFs and a chromatin modulator. RNA-seq data confirmed their elevated transcript levels in KM-H2 (**Fig 5A**). Afterwards, we performed siRNA-mediated knockdowns of these regulatory candidates, demonstrating their downregulation and their effects on TBX3 expression by RQ-PCR analysis (**Fig 5B**). The results showed that FOXC1 activated TBX3 strongly, KDM2B weakly while OTX2 showed no regulatory impact. FOXC1 has been reported as an aberrantly expressed TF in KM-H2 which is activated via genomic amplification at 6p25 [58]. Interestingly, the KDM2B locus is also located at 12q24 and forming part of that amplicon which also targets TBX3 (**Fig 5C**). Thus, both TBX3 itself and three identified activators, namely E2F4, FOXC1 and KDM2B, were rearranged by genomic amplification in KM-H2. Finally, analysis of expression profiling data from HL patients (GSE12453) indicated significantly elevated transcript levels of E2F4 and FOXC1 but not of KDM2B (**S4 Fig**). These results show clinical relevance for increased expression of E2F4 and FOXC1 in this malignancy.

### TBX3 represses CDKN2A, NFKBIB and CD19 in HL

**Fig 6:**
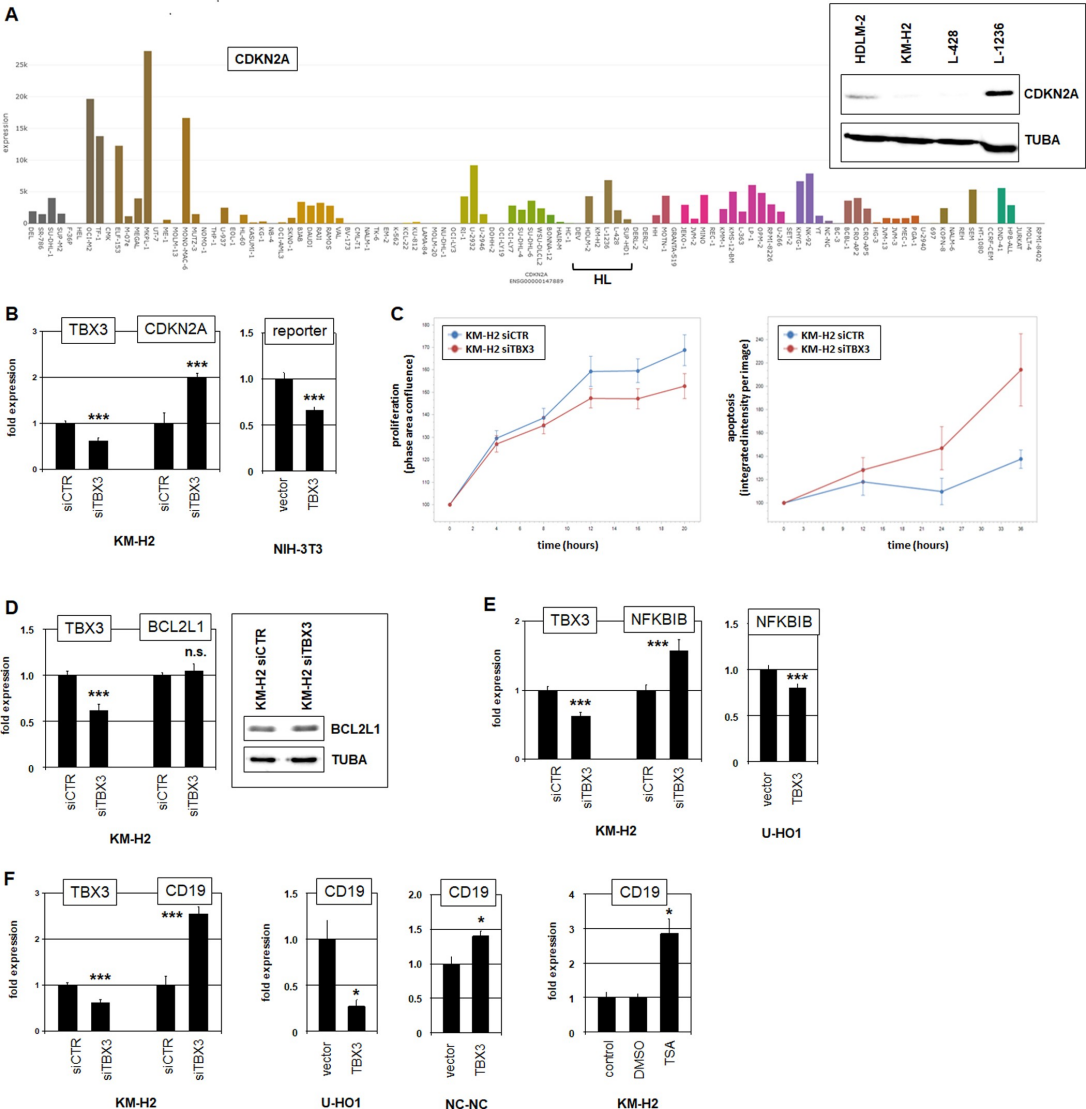
TBX3 represses CDKN2A, NFKBIB and CD19. (A) RNA-seq data show expression levels of CDKN2A in 100 leukemia/lymphoma cell lines using dataset E-MTAB-7721. Transcript levels of CDKN2A are very heterogenious, showing reduced gene activity in HL cell line KM-H2. HL cell lines are indicated. Western blot analysis of CDKN2A (insert) reflects transcript levels, showing elevated expression in L-1236, medium level in HDLM-2, and undetectable protein in KM-H2 and L-428. TUBA served as loading control. (B) RQ-PCR analysis of CDKN2A after siRNA-mediated knockdown of TBX3 (left). Data indicated that TBX3 inhibited CDKN2A expression. Reporter-gene assay in NIH-3T3 cells demonstrated that TBX3 suppressed CDKN2A via its consensus site located at –1293 bp (right). (C) Live-cell-imaging of KM-H2 cells treated for siRNA-mediated knockdown of TBX3 showed that TBX3 promoted proliferation (left, p = 0.01) and inhibited apoptosis (right, p = 0.04). (D) RQ-PCR analysis (left) and Western blot analysis (right) of BCL2L1/BCLXL after siRNA-mediated knockdown of TBX3. Data indicated that TBX3 regulated neither transcription nor splicing of BCL2L1. (E) RQ-PCR analysis of NFKBIB after siRNA-mediated knockdown of TBX3 in KM-H2 (left), and after forced expression of TBX3 in U-HO1 (right). Data indicated that TBX3 inhibited the expression of NFkB-signaling inhibitor NFKBIB. (F) RQ-PCR analysis of CD19 after siRNA-mediated knockdown of TBX3 in KM-H2 (left), and after forced expression of TBX3 in U-HO1 and NC-NC (middle). RQ-PCR analyses of CD19 after treatment with histone deacetylase inhibitor TSA in KM-H2 (right). The data indicated that TBX3 inhibited the transcription of B-cell differentiation marker CD19 by recruitment of histone deacetylases.

To identify potential target genes of TBX3 we reanalyzed our comparative expression profiling data (**S1 Table**). Because TBX3 has been described as repressor we looked for genes conspicuously downregulated in KM-H2 [59]. CDKN2A was among the top-100 silenced genes, and reportedly a TBX3 target in normal development and solid cancers [60–62]. CDKN2A encodes a cell cycle inhibitor and represents a tumor suppressor gene frequently inactivated in cancers by various mechanisms [63]. RNA-seq data and Western blot analysis confirmed silenced CDKN2A in KM-H2 (**Fig 6A**). SiRNA-mediated knockdown of TBX3 in KM-H2 raised CDKN2A expression, showing its suppressive role in HL (**Fig 6B**). Furthermore, an established reporter gene assay using an isolated genomic fragment of the CDKN2A promoter region from KM-H2 which contains a TBX3 consensus site at -1293 bp (AGGTGTGA) confirmed the repressive impact of TBX3 on this gene (**Fig 6B**). In addition, functional analysis by live-cell-imaging of TBX3 performed after its knockdown in KM-H2 cells showed significantly reduced proliferation (**Fig 6C**). Thus, TBX3 promoted proliferation in HL cell line KM-H2 probably via CDKN2A suppression. Additional live-cell imaging analysis of KM-H2 cells treated for siRNA-mediated knockdown indicated that TBX3 also inhibited apoptosis (**Fig 6C**). The same experimental setting was performed after knockdown of E2F4, demonstrating similar effects in proliferation and apoptosis which may be operated by its target TBX3 (**S5 Fig**).

To examine the underlying mechanism of TBX3 in mediation of apoptosis we focused on BCL2L1 and the NFkB-pathway. BCL2L1 (also called BCL-XL) encodes an anti-apoptotic protein of the BCL2-family which is regulated by alternative splicing, a process reportedly influenced by TBX3 [64]. However, TBX3-knockdown experiments and Western blot analysis discounted the regulatory impact of TBX3 in BCL2L1 transcription and splicing, respectively (**Fig 6D**). Activated NFkB-signaling represents a hallmark of HL. Many NFkB factors and their regulators are aberrantly expressed in HL and mediate survival of the malignant cells [41]. Here, we analyzed NFkB-inhibitor NFKBIB which has been reported as target of TBX3 in mammary glands [65]. Knockdown experiments in KM-H2 and forced expression in U-HO1 demonstrated that TBX3 inhibited the expression of NFKBIB in HL cells as well (**Fig 6E**). Thus, repression of NFkB-inhibitor NFKBIB by TBX3 forms an additional mechanism of aberrant NFkB-pathway activation in HL which may operate anti-apoptotically in KM-H2.

Suppression of B-cell differentiation via deregulation of developmental TFs is an additional hallmark of HL [38–40]. To examine the potential impact of TBX3 in B-cell differentiation we analyzed the expression of B-cell marker CD19 which is aberrantly downregulated in HL. RQ-PCR analysis demonstrated that TBX3 inhibited the transcription of CD19 in HL as indicated by TBX3 knockdown in KM-H2 or forced expression of TBX3 in U-HO1 (**Fig 6F**). However, flow cytometry data showed no alteration of CD19 expression at the protein level in knockdown-treated KM-H2 cells (**S6A Fig**). In contrast, forced expression of TBX3 in the CD19-positive normal B-cell line NC-NC resulted in slightly elevated expression of CD19 at the RNA level which was probably too weak for detection at the protein level (**Figs 6F and S6B**). These results show that the transcriptional regulation of CD19 differs between HL and normal B-cells. Therefore, the presence of particular cofactors may influence the activity of TBX3 in abnormal B-cells. Consistently, treatment of KM-H2 with histone deacetylase inhibitor TSA resulted in increased transcription of CD19 (**Fig 6F**), demonstrating an inhibitory impact of these chromatin-modifying cofactors. TF binding site analysis of the upstream regions of NFKBIB and CD19 revealed TBX consensus sequences at -3441 bp and -1758 bp, respectively, suggesting that TBX3 regulates the transcription these genes directly.

Finally, gene set annotation analysis of the top-1000 downregulated genes in KM-H2 versus control HL cell lines revealed several GO-terms, including regulation of growth, regulation of apoptotic signaling pathway, and regulation of B-cell differentiation (**S1 and S2 Tables**), supporting the above findings of oncogenic TBX3 activity in HL. Taken together, our functional data indicate that TBX3 activated proliferation and NFkB-signaling and inhibited differentiation, and is thus to be regarded as a potent oncogene in HL subsets.

## Discussion

Comprehensive expression analysis of T-box genes in early hematopoiesis and lymphopoiesis revealed a specific gene signature therein which we have termed TBX-code (**Fig 1**). This code covers physiological activities for six T-box genes and serves to illuminate regulatory processes in hematopoiesis. Furthermore, the TBX-code allows the identification of novel T-box oncogenes which may prove useful for diagnostics and tenable identification of therapeutic targets in lymphoid malignancies.

The TBX-code revealed normal expression of all six T-box gene members in T-cell development, representing the highest plurality of these genes in lymphopoiesis. In contrast, just two T-box genes were detected in developing B-cells. Accordingly, T-box gene activity was absent in germinal centre and plasma B-cells as well as in ILC2 and ILC3. MGA expression was detected in stem and progenitor cells, supporting reported functions of this gene in stemness and survival of pluripotent stem cells [66–68]. Furthermore, our data are consistent with roles already described for EOMES and TBX21 in B-cells, NK-cells, T-cells and ILC1 [26,28,29,34,69]. Thus, we uncovered novel expression profiles of T-box genes and observed basic concordances between the data of this established TBX-code and the literature.

Gene codes describe expression patterns of related genes in particular tissues or body regions. The NKL- and TALE-codes represent particular subgroups of homeobox genes. They contribute to the understanding of normal developmental processes in hematopoiesis and served as platforms for identification and evaluation of deregulated homeobox genes in lymphoid and myeloid malignancies [13,24]. Thus, the TBX-code enabled the identification of deregulated T-box genes in HL and T-ALL and indicated that these genes play no obvious oncogenic role in ALCL. These findings may reflect the normal T-box gene activities detected in the cells of origin for these tumors. ALCL reportedly derives from the ILC3 entity which does not express any T-box gene [36]. This implies that T-box gene activity impacts processes inimical to ILC3 differentiation [34]. In contrast, T-ALL cells aberrantly express several T-box genes and derive from thymocytes which physiologically express the highest number of T-box genes [35]. Therefore, T-box genes may control key processes in developing T-cells which are prone to malignant transformation. This logic may also apply to NKL homeobox genes: DN-thymocytes express a high number of NKL homeobox genes and in T-ALL these genes represent the largest group of oncogenes [13]. However, HRS cells aberrantly express seven T-box genes and derive from GC B-cells which are negative for T-box gene activities [37]. Therefore, additional investigation is required to interpret the relationships between physiological and aberrant expression patterns of related developmental factors.

**Fig 7:**
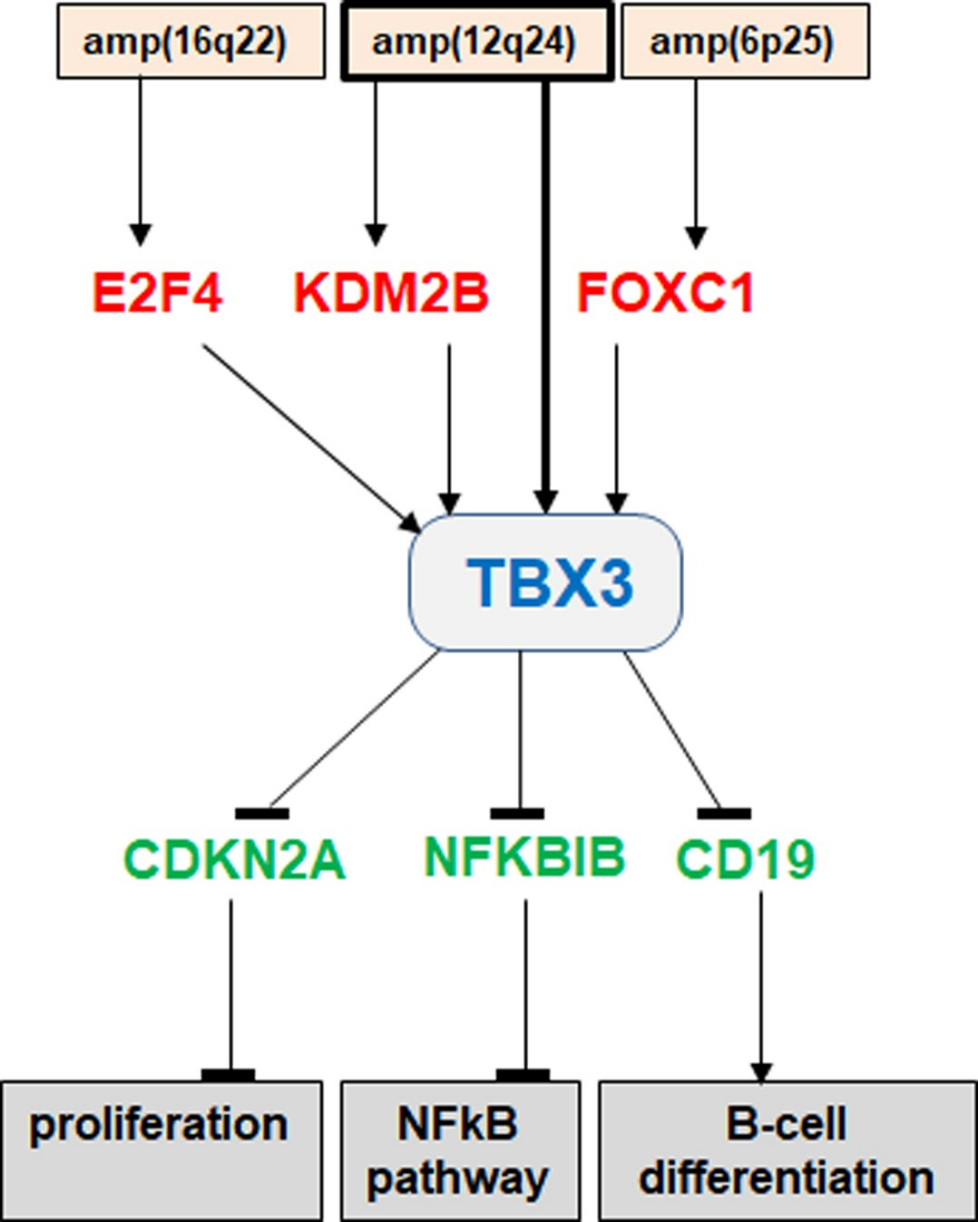
Gene regulatory network around TBX3 in HL. This diagram summarizes the results of this study, placing TBX3 at the centre of an aberrant gene regulatory network. TBX3 is directly targeted by genomic amplification just like its activators E2F4, KDM2B and FOXC1. TBX3 inhibits expression of CDKN2A, NFKBIB and CD19, thereby impacting proliferation, NFkB-signaling and B-cell differentiation, respectively.

In this study we focused on ectopically activated TBX3 in HL. TBX3 belongs to the Tbx2 subfamiliy which lies outside the lymphoid TBX-code. Normally, TBX3 is involved in the development of multiple tissues and organs, like breast, limb, lung and retina [54,70,71]. Accordingly, mutation or deregulation of TBX3 underlies developmental diseases, like the Ulnar-mammary syndrome [14,54]. In addition, TBX3 is reportedly deregulated in several cancers including breast, lung and melanoma [54]. Using HL cell line KM-H2 as model we identified three oncogenic TBX3 activators, namely E2F4, FOXC1 and KDM2B as summarized in **Fig 7**. OTX2 showed no impact on TBX3 expressiion although both genes are coexpressed and their regulation is connected in developing eyes [72]. We found that TBX3 and its identified activators are targeted by genomic amplification in KM-H2. The amplicons at 12q24, containing TBX3 and KDM2B, and at 16q22, containing E2F4, are recurrently targeted in HL, highlighting the potential activation of these genes in patients [73]. In contrast, targeted amplification of FOXC1 at 6p25 has been described in KM-H2 previously, however, no copy number gains have been reported for this chromosomal position so far in primary HL samples, indicating that malignant activation of FOXC1 mostly conducted non cytogenetically [73–75]. Finally, E2F4, FOXC1 and KDM2B play oncogenic roles in other types of cancer as well [76–78], supporting their identified pathogenic potential in HL.

The subcellular distribution of TBX3 protein in KM-H2 cells showed a uniform distribution in the cytoplasma and speckled concentrations in the nucleus. This pattern indicated that the TF TBX3 regulates specific nuclear target genes and is itself controlled by intracellular transport. TBX3 is a transcriptional repressor which recruits polycomb repressor complex (PRC)2 and HDACs to inhibit its gene targets [59,79]. PRCs are found in speckles or nuclear bodies as well, indicating that TBX3 is part of such nuclear repressor complexes in KM-H2 [80]. Interestingly, TBX3 shares repressive gene activity with TBX-code member MGA which binds and recruits PRC1.6 to inhibit lineage-specific genes in early embryonal development [81]. This repressive function may underlie the pathogenic role for ectopically expressed TBX3 in HL. The physiological subcellular localization of TBX21 is regulated by PD1 in T-cells and aberrant enhancement of nuclear import from the cytoplasm has been reported for TBX2 in breast cancer cells, indicating that this type of regulation may play a role for the related TBX3 as well [82,83]. Thus, data from the literature support the interpretation of our observed subcellular pattern of TBX3 protein which may reflect its regulatory conditions.

According to its documented role as transcriptional repressor, we report for TBX3 in HL cell line KM-H2 three suppressed genes bearing consensus T-box binding sites in their upstream regions. The identified target genes CDKN2A, NFKBIB and CD19 are displayed in summarizing **Fig 7**. Furthermore, our data indicate that TBX3 performs these repressive impacts by recruitment of histone deacetylases, as demonstrated in breast cancer cells [84]. Functionally, target gene CDKN2A encodes an inhibitor of the cell cycle, NFKBIB an inhibitor of the NFkB-pathway, and CD19 a component of the B-cell receptor complex serving as marker for differentiated B-cells [63,85,86]. The tasks performed by these genes represent dysregulated hallmarks in HL. The oncogenic potential of TBX3 in suppressing cell differentiation may be related to its reported function in pluripotency and self-renewal, possibly by substituting silenced TBX-code member MGA [81,87,88]. Thus, while TBX3 manifests as a prominent oncogene in HL, follow-up studies are needed to gauge its usefulness as diagnostic marker and/or therapeutic target.

Taken together, we have established the TBX-code for early hematopoiesis and lymphopoiesis and used it to evaluate aberrant T-box gene activities in the lymphoid malignancies HL, ALCL and T-ALL. We have shown TBX3 to be ectopically expressed in HL and documented its role as a potent oncogene in subsets of patients.

## Supporting information

S1 Fig T-box gene screening in early hematopoiesis and lymphopoiesis. Analyses of five public datasets revealed activities of particular T-box genes in early lymphopoiesis, T-cell and B-cell development, mature lymphocytes, and mature and progenitor ILCs, using (A) RNA-seq dataset GSE69239 for HSC, LMPP, CLP, BCP, DN T-cells, DP T-cells, CD4+ T-cells and CD8+ T-cells, (B) gene expression profiling dataset GSE56315 for naïve, germinal centre, memory and plasma B-cells, (C) gene expression profiling dataset GSE72642 for NK-cells, CD4+ T-cells, CD8+ T-cells, and B-cells, and (D) RNA-seq datasets GSE124474 and E-MTAB-8494 for ILC1, ILC2, ILC3 and ILCP.
(TIF)

S2 Fig T-box gene screening in HL, T-ALL and ALCL patients. (A) Analysis of T-box gene activities in HL patients using expression profiling datasets GSE12453 revealed six genes overexpressed in subsets of HL patients (red). Normal B-cells serve as controls and are indicated. (B) Analysis of T-box gene activities in T-ALL patients using expression profiling datasets GSE26713 revealed six genes overexpressed in subsets of T-ALL patients (red). (C) Analysis of T-box gene activities in ALCL patients using expression profiling datasets GSE14879 showed absence of deregulated genes in ALCL patients.
(TIF)

S3 Fig T-box gene activity in leukemia/lymphoma cell lines (LL-100). Transcript levels of 16 T-box genes in 100 leukemia/lymphoma cell lines using RNA-seq dataset E-MTAB-7721. Note, TBX22 is not expressed and therefore omitted. HL cell lines are indicated.
(TIF)

S4 Fig Comparative gene expression profiling analysis of HL patients (GSE12453). Transcript levels of TBX3-activators E2F4, FOXC1 and KDM2B in HL patients were compared to normal B-cell entities as controls and are visualized as boxplots. Statistical significance was calculated by T-test. The according p-values are indicated, showing significant differences for E2F4 and FOXC1.
(TIF)

S5 Fig Life-cell-imaging analyses of KM-H2 treated for E2F4 knockdown. Live-cell-imaging of KM-H2 cells treated for siRNA-mediated knockdown of E2F4 showed that E2F4 promoted proliferation (left, p = 0.035) and inhibited apoptosis (right, p = 0.001).
(TIF)

S6 Fig Flow cytometry analysis of CD19. (A) Flow cytometry analysis of CD19 in KM-H2 treated for TBX3 knockdown. (B) Flow cytometry analysis of CD19 in NC-NC treated for TBX3 overexpression.
(PDF)

S7 Fig (TIF)

S8 Fig (TIF)

S9 Fig (TIF)

S10 Fig (TIF)

S11 Fig (TIF)

S12 Fig (TIF)

S13 Fig (TIF)

S14 Fig (TIF)

S1 Table Comparative gene expression profiling analysis of HL cell lines (GSE115191). Gene expression profiling data of eight HL cell lines are indicated, highlighting KM-H2 by a gray background. Line DH451SU gives medium expression levels for control cell lines DEV, HDLM-2, L-428, L-540, L-1236, SUP-HD1 and U-HO1. Line K-DH451SU gives the calculated differential gene expression levels between KM-H2 and the controls.
(XLS)

S2 Table GO-term analysis of top-1000 downregulated genes in KM-H2. Expression profiling analysis of HL cell line KM-H2 in comparison to seven control cell lines revealed differentially expressed genes (see S1 Table). The top-1000 downregulated genes in KM-H2 were analyzed using the DAVID online tool generating a list of GO terms. This list indicates deregulated functions in KM-H2, including regulation of growth, regulation of B-cell differentiation, and regulation of apoptotic signaling pathways (arrow heads).
(TIF)
