# Supplementary figures and images for: Establishment of the TBX-code reveals aberrantly activated T-box gene TBX3 in Hodgkin lymphoma
Source: PLoS One. 2021 Nov 22;16(11):e0259674. doi: 10.1371/journal.pone.0259674 (PMC8608327; doi:10.1371/journal.pone.0259674)

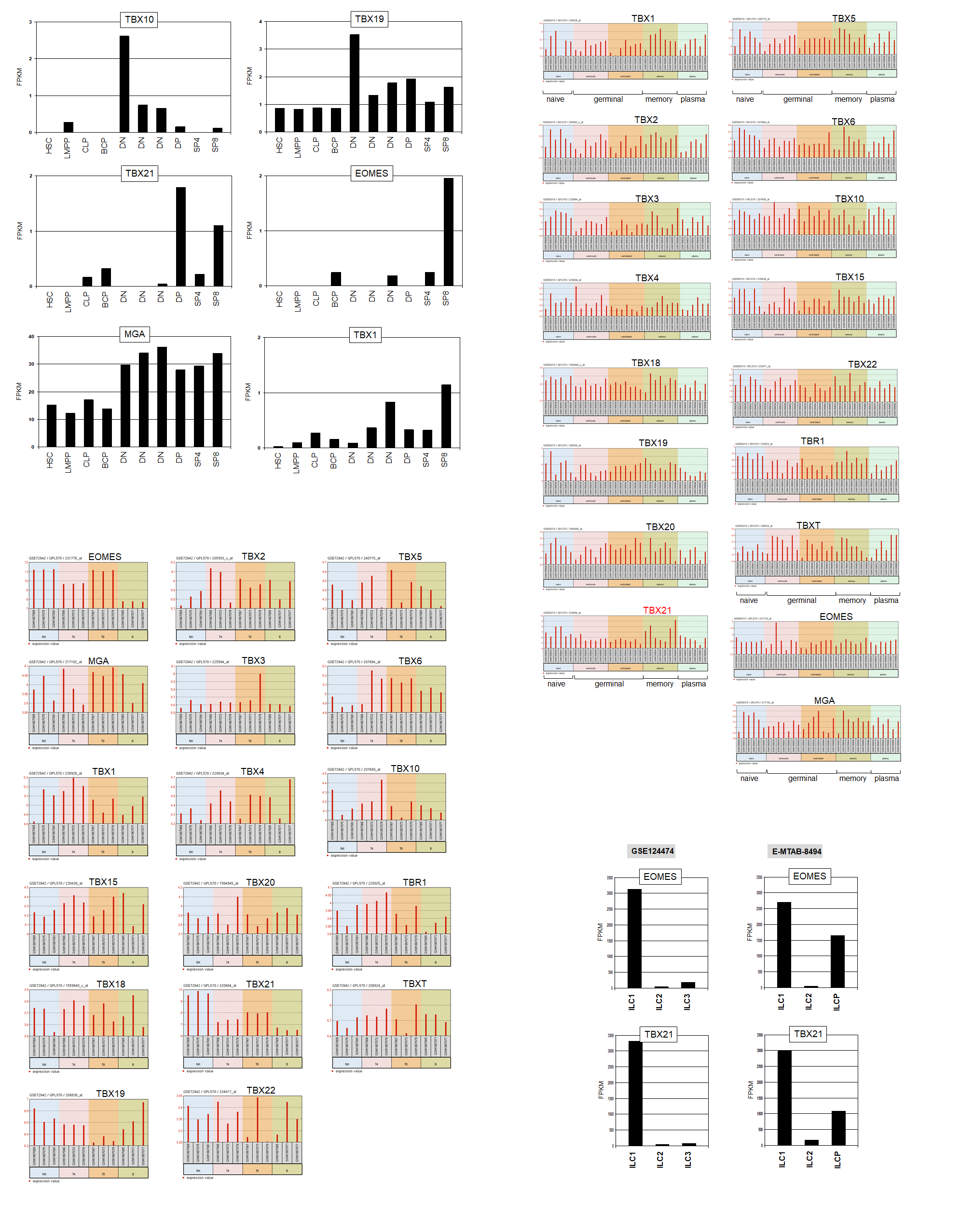

Supplement: S1 Fig — Analyses of five public datasets revealed activities of particular T-box genes in early lymphopoiesis, T-cell and B-cell development, mature lymphocytes, and mature and progenitor ILCs, using (A) RNA-seq dataset GSE69239 for HSC, LMPP, CLP, BCP, DN T-cells, DP T-cells, CD4+ T-cells and CD8+ T-cells, (B) gene expression profiling dataset GSE56315 for naïve, germinal centre, memory and plasma B-cells, (C) gene expression profiling dataset GSE72642 for NK-cells, CD4+ T-cells, CD8+ T-cells, and B-cells, and (D) RNA-seq datasets GSE124474 and E-MTAB-8494 for ILC1, ILC2, ILC3 and ILCP. (TIF) [file pone.0259674.s001.tif]

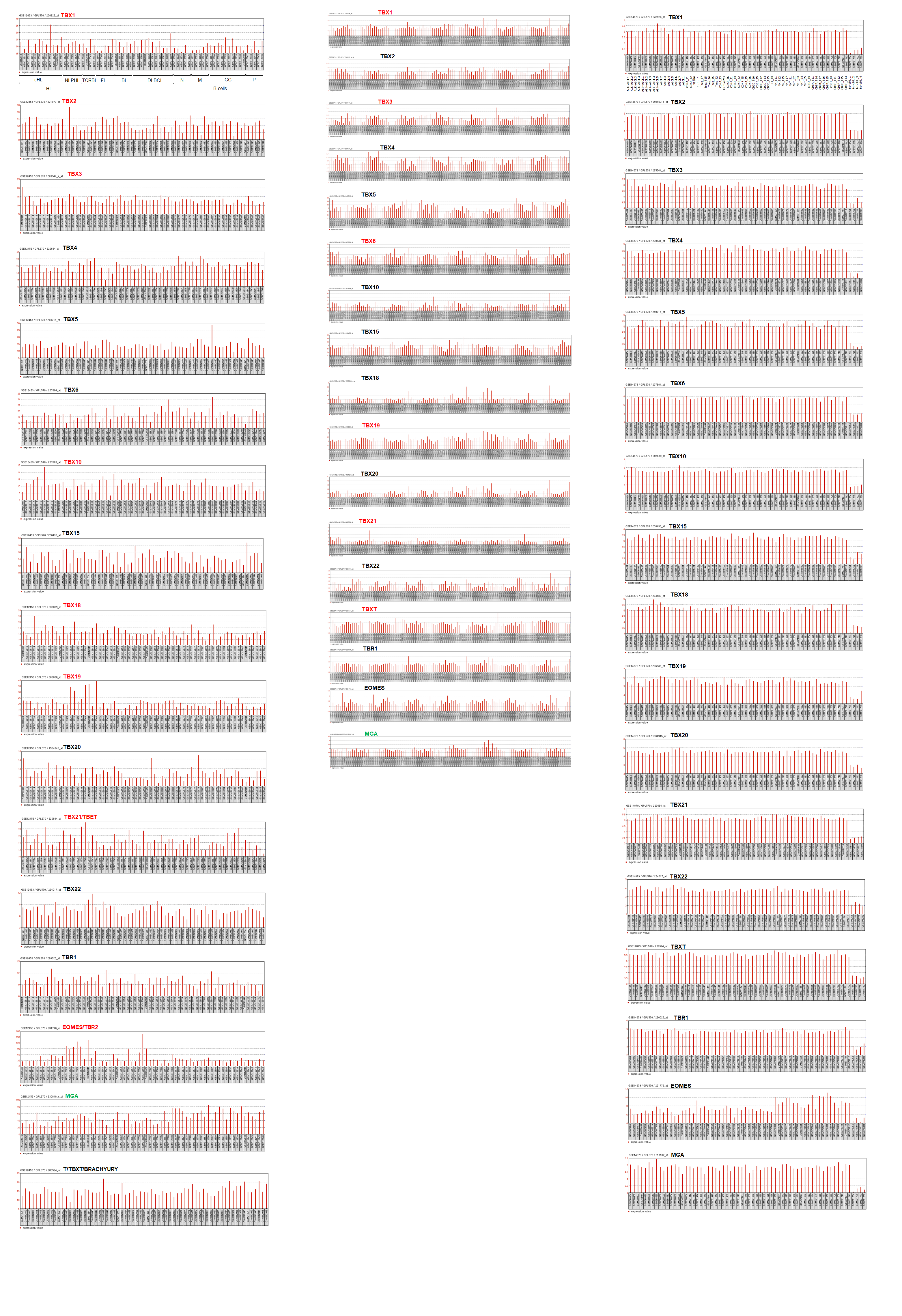

Supplement: S2 Fig — (A) Analysis of T-box gene activities in HL patients using expression profiling datasets GSE12453 revealed six genes overexpressed in subsets of HL patients (red). Normal B-cells serve as controls and are indicated. (B) Analysis of T-box gene activities in T-ALL patients using expression profiling datasets GSE26713 revealed six genes overexpressed in subsets of T-ALL patients (red). (C) Analysis of T-box gene activities in ALCL patients using expression profiling datasets GSE14879 showed absence of deregulated genes in ALCL patients. (TIF) [file pone.0259674.s002.tif]

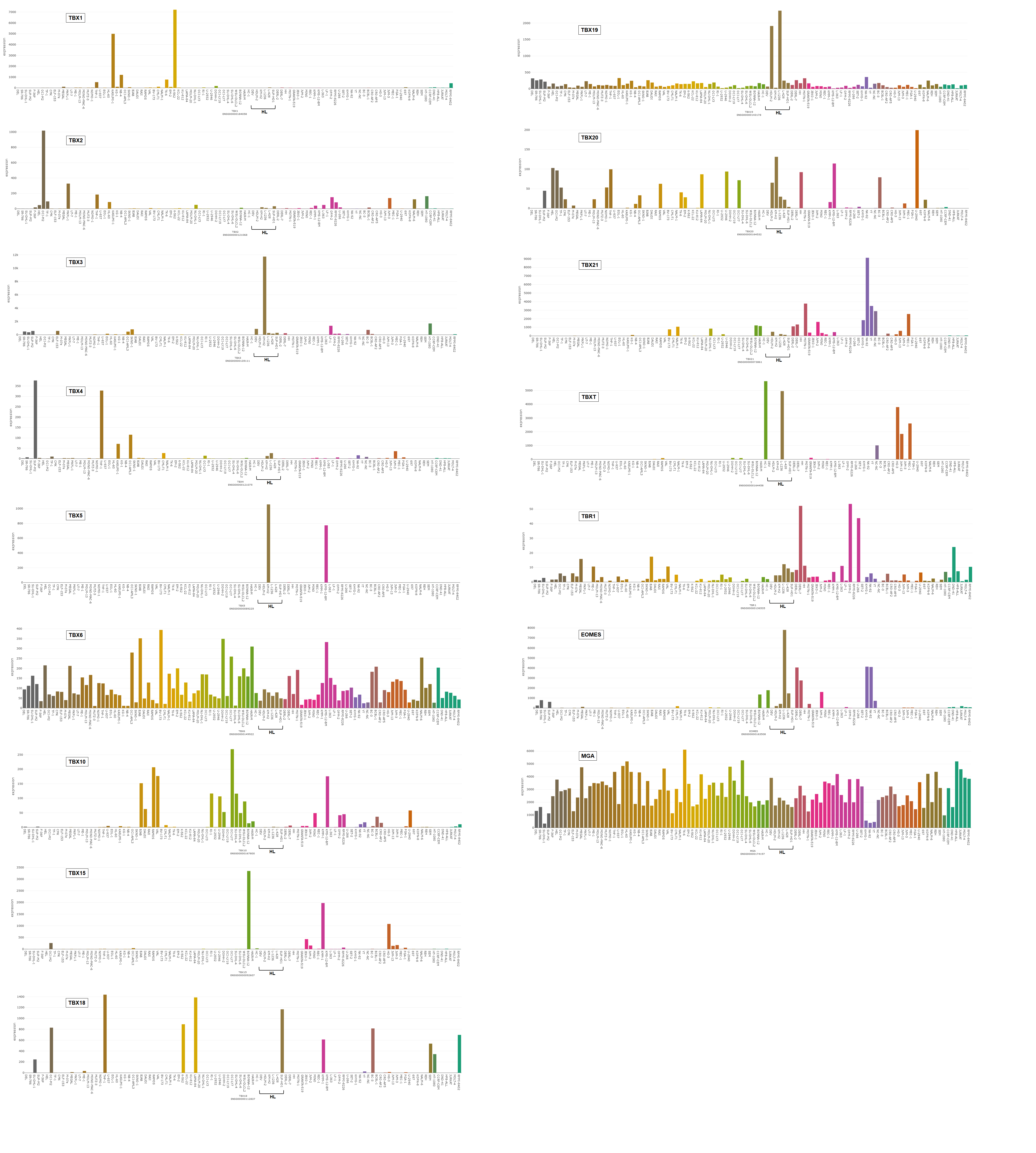

Supplement: S3 Fig — Transcript levels of 16 T-box genes in 100 leukemia/lymphoma cell lines using RNA-seq dataset E-MTAB-7721. Note, TBX22 is not expressed and therefore omitted. HL cell lines are indicated. (TIF) [file pone.0259674.s003.tif]

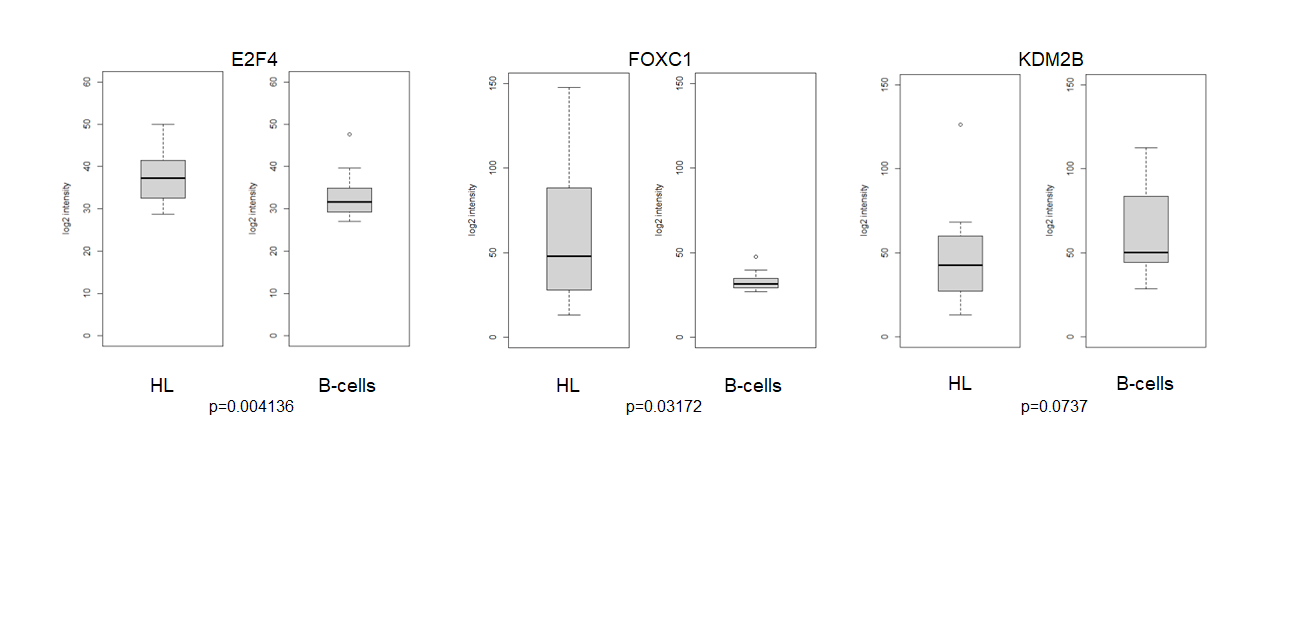

Supplement: S4 Fig — Transcript levels of TBX3-activators E2F4, FOXC1 and KDM2B in HL patients were compared to normal B-cell entities as controls and are visualized as boxplots. Statistical significance was calculated by T-test. The according p-values are indicated, showing significant differences for E2F4 and FOXC1. (TIF) [file pone.0259674.s004.tif]

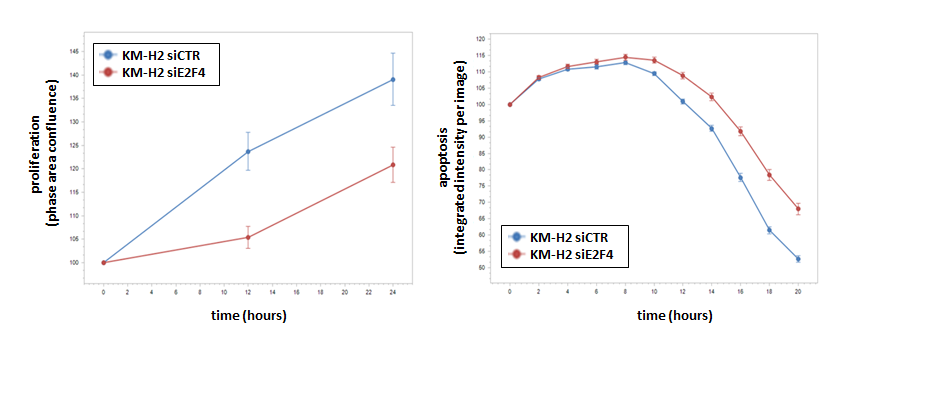

Supplement: S5 Fig — Live-cell-imaging of KM-H2 cells treated for siRNA-mediated knockdown of E2F4 showed that E2F4 promoted proliferation (left, p = 0.035) and inhibited apoptosis (right, p = 0.001). (TIF) [file pone.0259674.s005.tif]

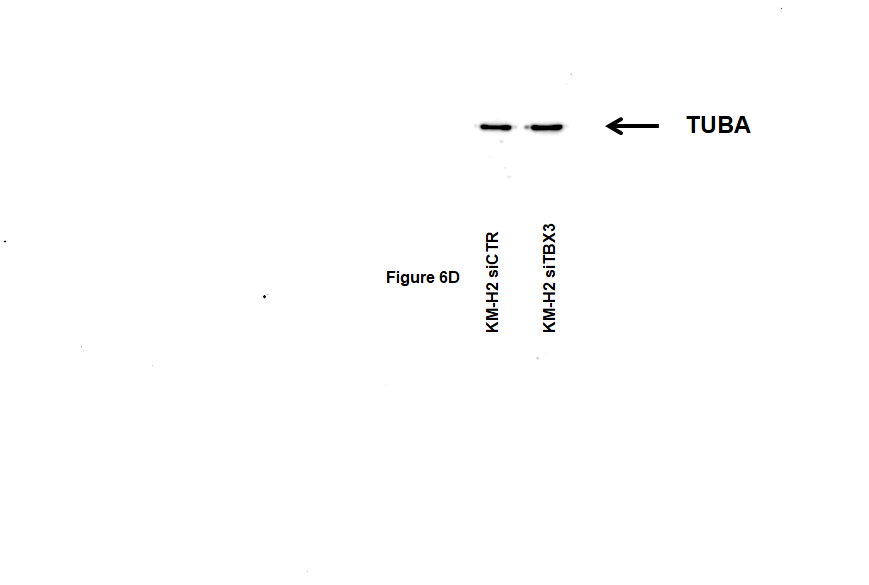

Supplement: S7 Fig — (TIF) [file pone.0259674.s007.tif]

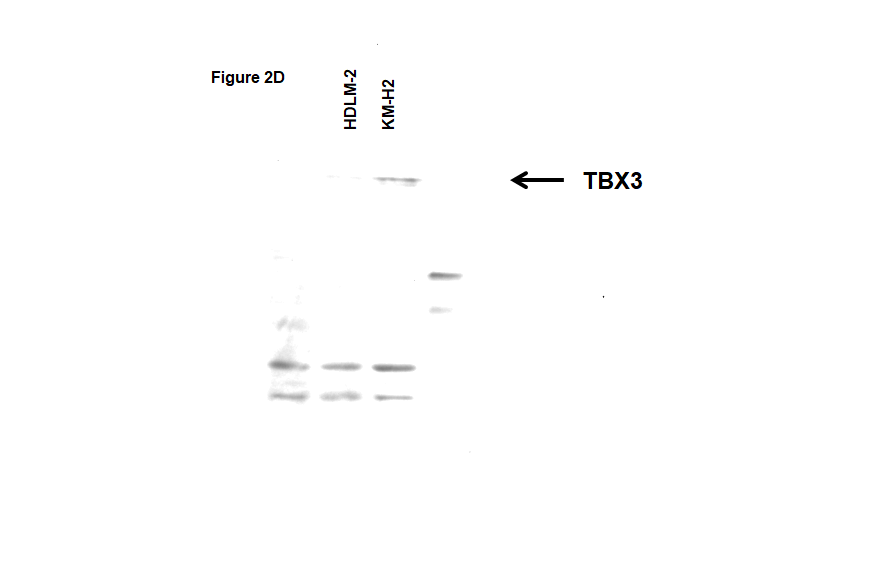

Supplement: S8 Fig — (TIF) [file pone.0259674.s008.tif]

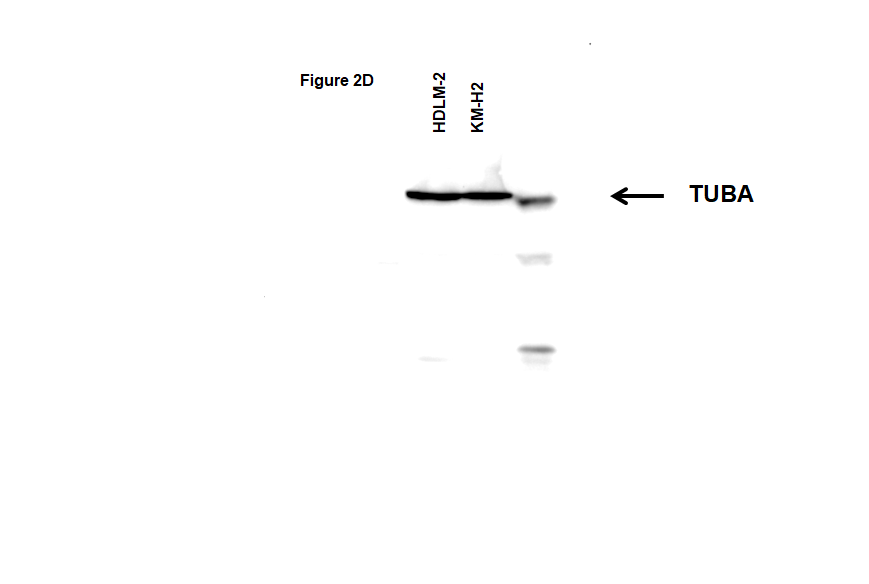

Supplement: S9 Fig — (TIF) [file pone.0259674.s009.tif]

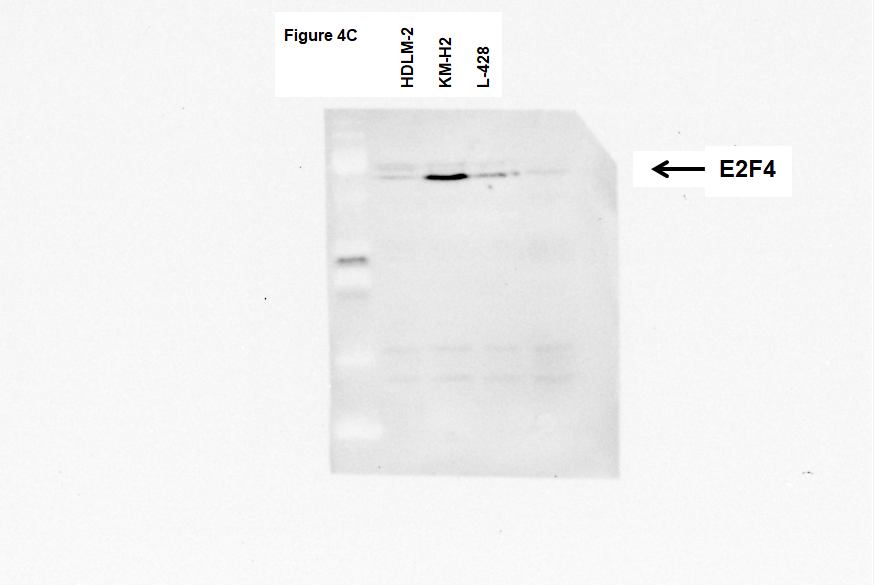

Supplement: S10 Fig — (TIF) [file pone.0259674.s010.tif]

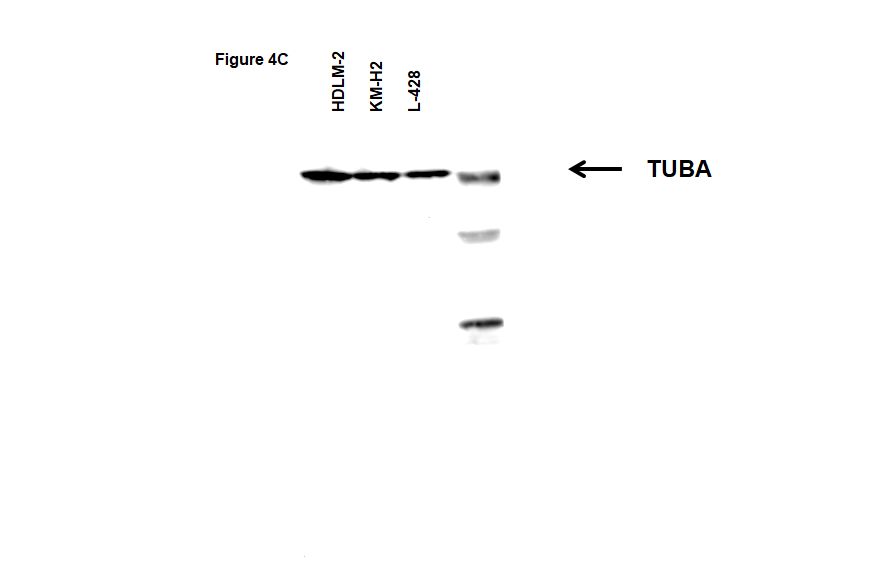

Supplement: S11 Fig — (TIF) [file pone.0259674.s011.tif]

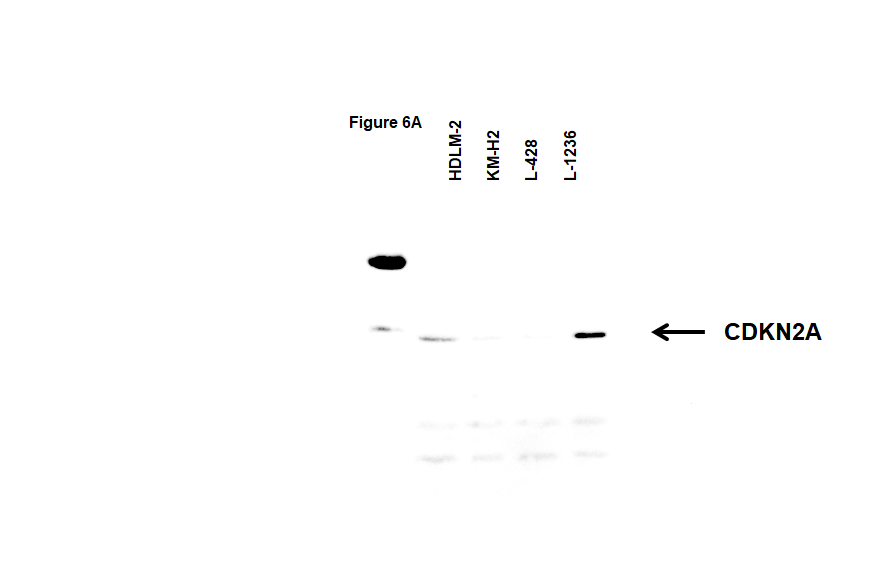

Supplement: S12 Fig — (TIF) [file pone.0259674.s012.tif]

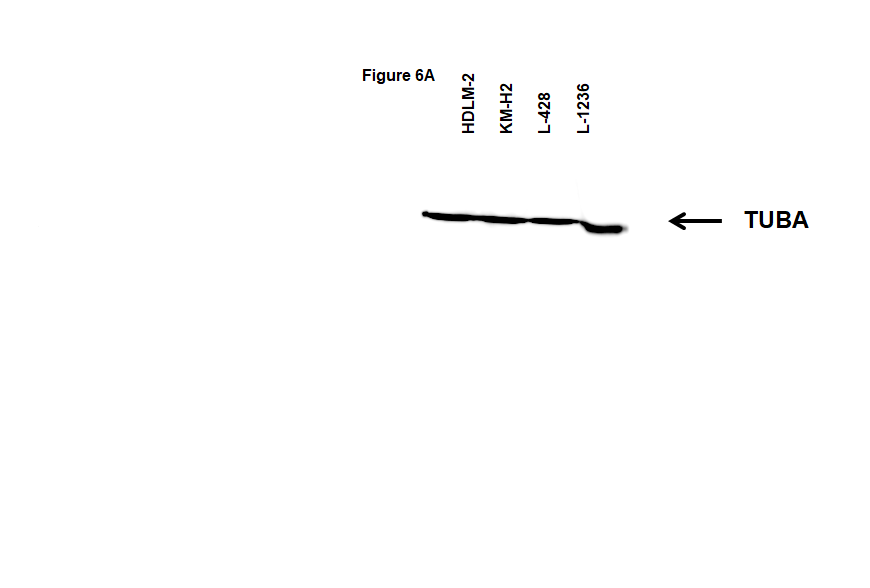

Supplement: S13 Fig — (TIF) [file pone.0259674.s013.tif]

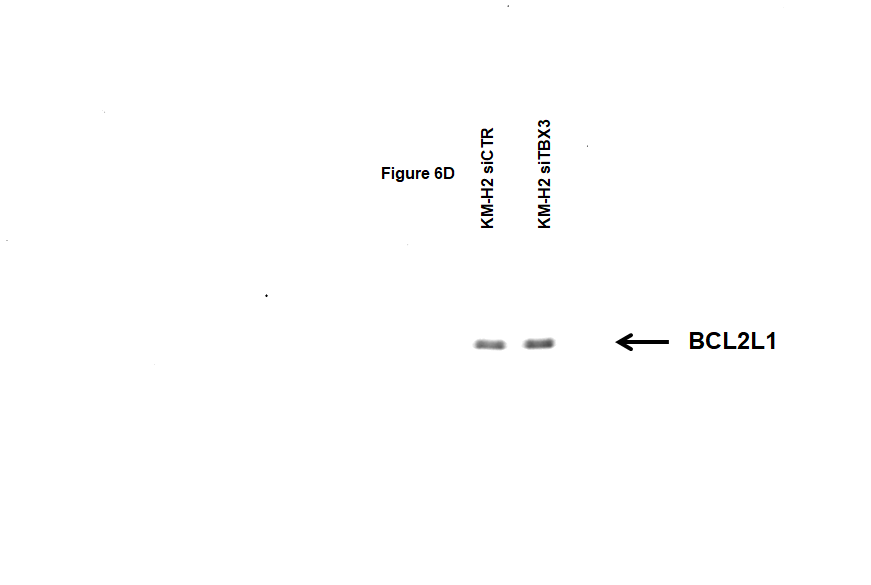

Supplement: S14 Fig — (TIF) [file pone.0259674.s014.tif]

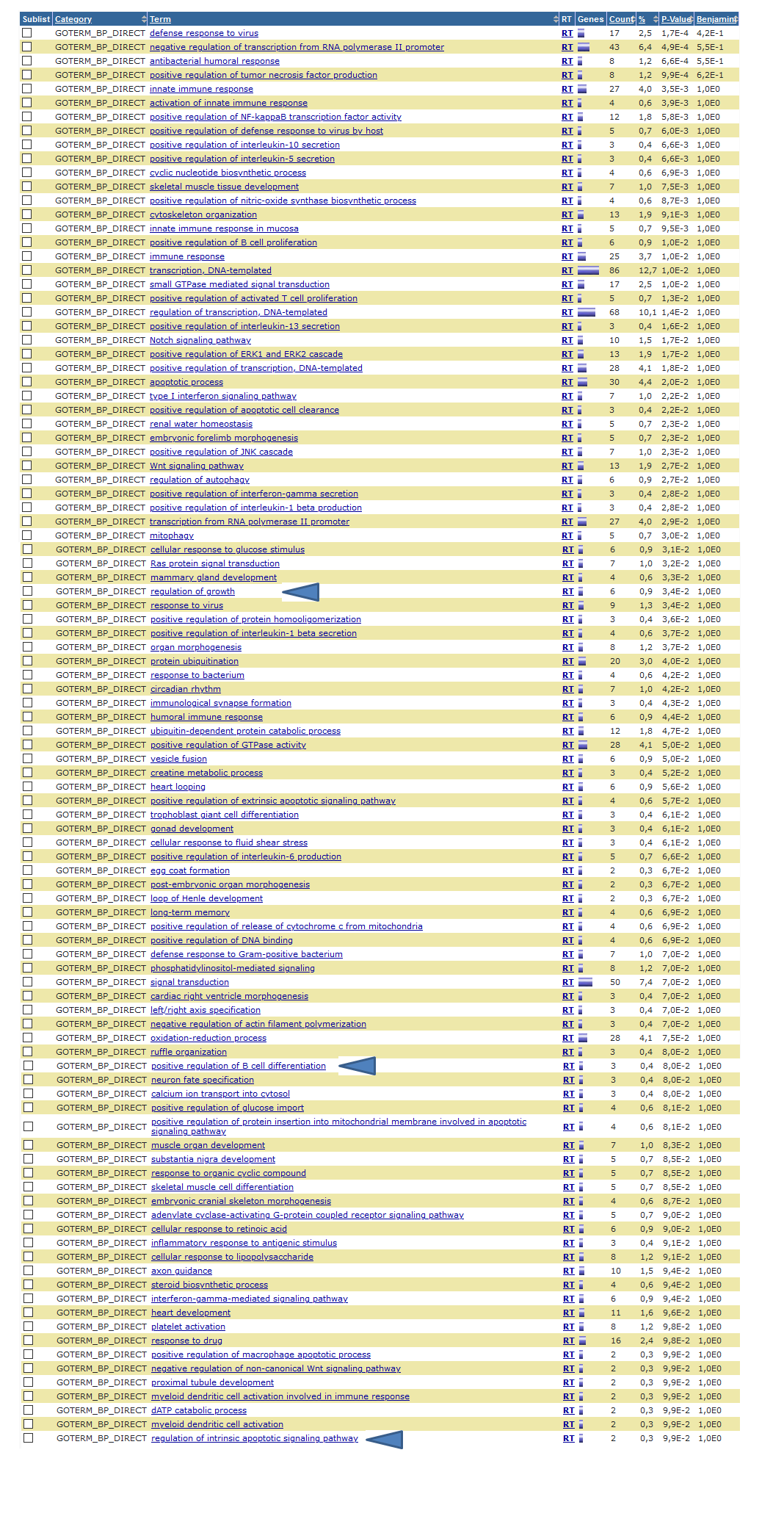

Supplement: S2 Table — Expression profiling analysis of HL cell line KM-H2 in comparison to seven control cell lines revealed differentially expressed genes (see S1 Table). The top-1000 downregulated genes in KM-H2 were analyzed using the DAVID online tool generating a list of GO terms. This list indicates deregulated functions in KM-H2, including regulation of growth, regulation of B-cell differentiation, and regulation of apoptotic signaling pathways (arrow heads). (TIF) [file pone.0259674.s016.tif]
