# Supplementary material for: Establishment of the TBX-code reveals aberrantly activated T-box gene TBX3 in Hodgkin lymphoma
Source: PLoS One. 2021 Nov 22;16(11):e0259674. doi: 10.1371/journal.pone.0259674 (PMC8608327; doi:10.1371/journal.pone.0259674)
Supplement: S6 Fig — (A) Flow cytometry analysis of CD19 in KM-H2 treated for TBX3 knockdown. (B) Flow cytometry analysis of CD19 in NC-NC treated for TBX3 overexpression. (PDF) [file pone.0259674.s006.pdf]

A

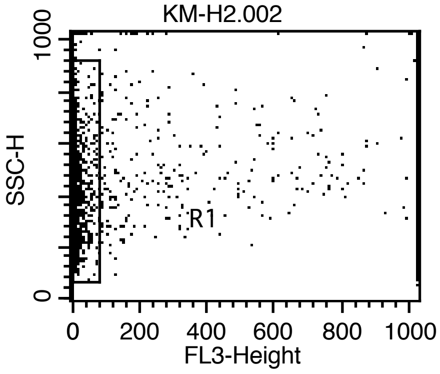

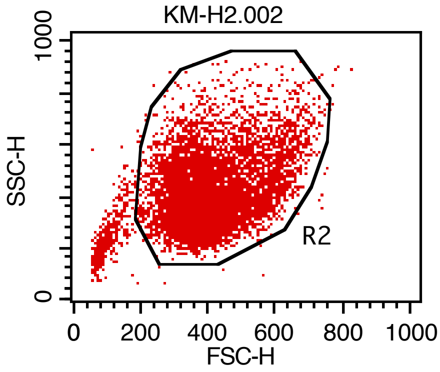

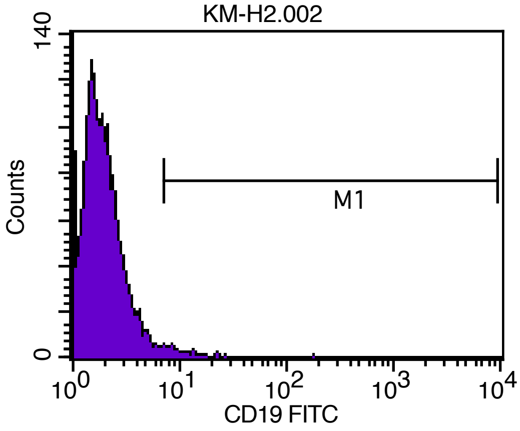

File: KM-H2.002
Tube: CD19
Acquisition Date: 20-Aug-21

Marker	% Gated
All	100.00
M1	1.05

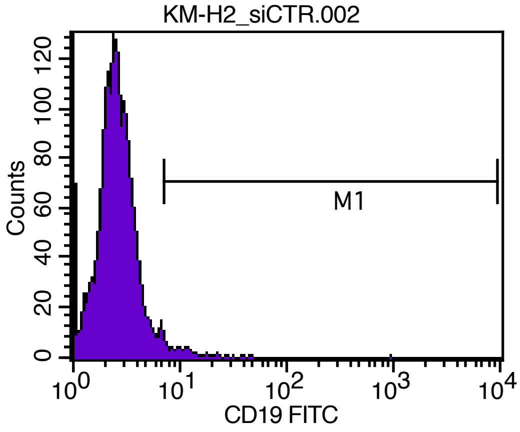

File: KM-H2_siCTR.002
Tube: CD19
Acquisition Date: 20-Aug-21

Marker	% Gated
All	100.00
M1	1.45

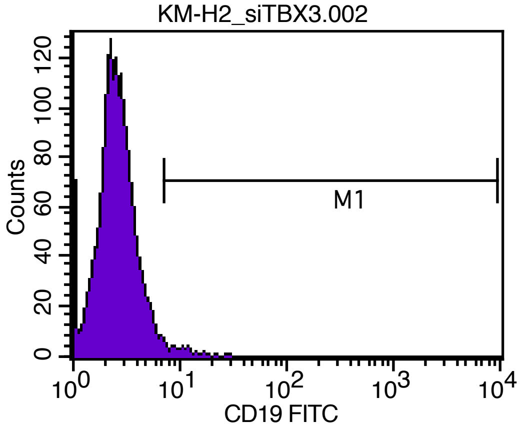

File: KM-H2_siTBX3.002
Tube: CD19
Acquisition Date: 20-Aug-21

Marker	% Gated
All	100.00
M1	1.46

B

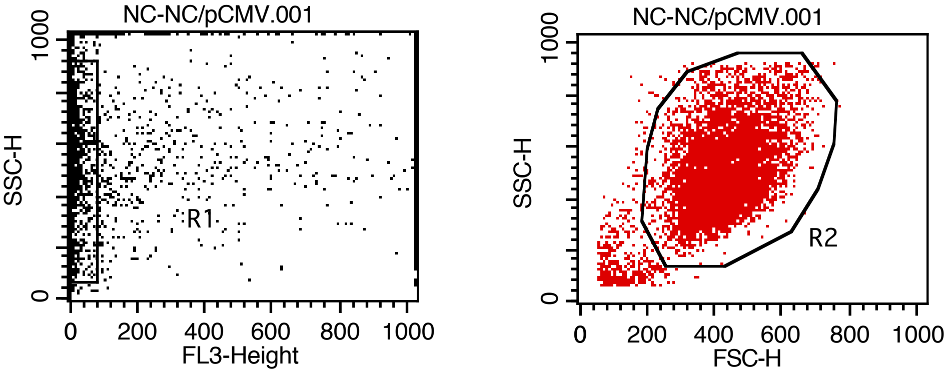

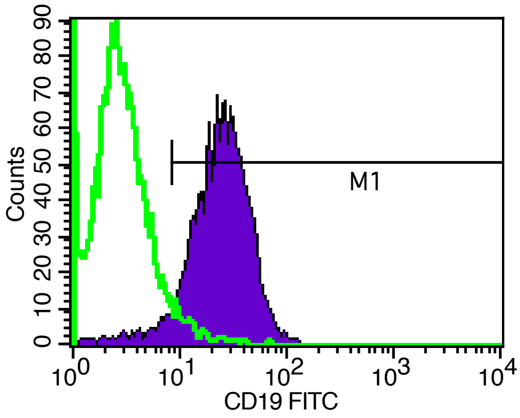

File: NC-NC.005
Tube: CD19
Acquisition Date: 26-Aug-21

Marker	% Gated
All	100.00
M1	95.89

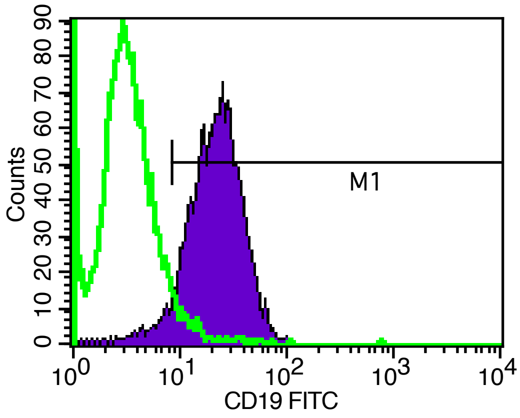

File: NC-NC/pCMV.005
Tube: CD19
Acquisition Date: 26-Aug-21

Marker	% Gated
All	100.00
M1	95.31

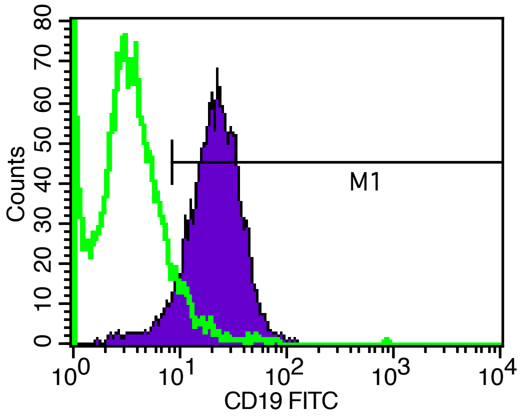

File: NC-NC/TBX3.005
Tube: CD19
Acquisition Date: 26-Aug-21

Marker	% Gated
All	100.00
M1	95.35
